# Capsaicin Inhibits *Shigella flexneri* Intracellular Growth by Inducing Autophagy

**DOI:** 10.3389/fphar.2022.903438

**Published:** 2022-07-06

**Authors:** Priyanka Basak, Priyanka Maitra, Uzma Khan, Kalyani Saha, Satya Sundar Bhattacharya, Moumita Dutta, Sushmita Bhattacharya

**Affiliations:** ^1^ Division of Biochemistry, National Institute of Cholera and Enteric Diseases, Kolkata, India; ^2^ Department of Environmental Science, Tezpur University, Tezpur, India; ^3^ Division of Electron Microscopy, National Institute of Cholera and Enteric Diseases, Kolkata, India

**Keywords:** *Shigella flexneri*, autophagy, TFEB, capsaicin, gene transcription

## Abstract

Antibiotic treatment plays an essential role in preventing *Shigella* infection. However, incidences of global rise in antibiotic resistance create a major challenge to treat bacterial infection. In this context, there is an urgent need for newer approaches to reduce *S. flexneri* burden. This study largely focuses on the role of the herbal compound capsaicin (Caps) in inhibiting *S. flexneri* growth and evaluating the molecular mechanism behind bacterial clearance. Here, we show for the first time that Caps inhibits intracellular *S. flexneri* growth by inducing autophagy. Activation of autophagy by Caps is mediated through transcription factor TFEB, a master regulator of autophagosome biogenesis. Caps induced the nuclear localization of TFEB. Activation of TFEB further induces the gene transcription of autophagosomal genes. Our findings revealed that the inhibition of autophagy by silencing TFEB and Atg5 induces bacterial growth. Hence, Caps-induced autophagy is one of the key factors responsible for bacterial clearance. Moreover, Caps restricted the intracellular proliferation of *S. flexneri*-resistant strain. The efficacy of Caps in reducing *S. flexneri* growth was confirmed by an animal model. This study showed for the first time that *S. flexneri* infection can be inhibited by inducing autophagy. Overall observations suggest that Caps activates TFEB to induce autophagy and thereby combat *S. flexneri* infection.

## 1 Introduction

*Shigella flexneri*, a common gastrointestinal pathogen, causes bacillary dysentery that affects millions every year worldwide (Nandy et al., 2011; Taneja and Mewara, 2016; Williams and Berkley, 2018; McQuade et al., 2020). According to the available information, *S. flexneri* infection is highly predominant in developing nations. Children below 5 years of age are affected with severe morbidity and mortality (Chang et al., 2016; Baker and The, 2018; Khalil et al., 2018; Chanin et al., 2019; Chen et al., 2020). The pathogen invades the intestinal epithelial and immune cells, causing ulcerative lesions in the gut under severe conditions (Killackey et al., 2016). To date, the only treatment option is antibiotics as no licensed vaccine has been developed (Hosangadi et al., 2019). Recently, the reverse vaccinology approach has been considered to improve vaccine research against *Shigella* (Hajialibeigi et al., 2021). Excessive use of antibiotics has led to a significant rise in antibiotic-resistant bacteria (Puzari et al., 2018; Ranjbar and Farahani, 2019). Hence, in the current scenario, there is an imperative need to search for alternative approaches in treating *S. flexneri* infection.

Presently, host-directed therapy is considered as an alternative therapeutic approach for the eradication of invasive pathogens (Rubinsztein et al., 2012; Kaufmann et al., 2018). Host-directed therapeutics can target the disease-causing signaling pathways and help eradicate microbes (Noad et al., 2017; Young et al., 2020). Autophagy plays a significant role among different host response mechanisms in combating infectious pathogens. Autophagy is a self-degradation mechanism used to eliminate unwanted materials like pathogens from the cell. Exploitation of autophagy has emerged as a new approach toward intracellular pathogens like *Mycobacterium tuberculosis* and *Salmonella typhimurium* (Kim et al., 2012; Ammanathan et al., 2020). In the context of infectious diseases, autophagy inducers such as vitamin D and antibiotics isoniazid and bedaquiline are reported to inhibit the proliferation of *Mycobacterium tuberculosis* and *H. pylori* (Yuk et al., 2009; Hu et al., 2019; Kim et al., 2019; Giraud-Gatineau et al., 2020). Similar strategies have also been used against viruses like HIV (Jo et al., 2013; Sharma et al., 2018). However, there are no such interventions reported against *S. flexneri*.

As autophagy is an important host defense mechanism occurring within the cell to clear pathogens, *S. flexneri* has evolved multiple ways to prevent autophagic recognition (Ogawa et al., 2005; Ra et al., 2016). *S. flexneri* circumvents autophagic degradation by releasing effector proteins (icsB and virA) and establishes itself in the intracellular niche of the colonic environment. Moreover, it has been reported earlier that Atg4b mutant cells inhibited *S. flexneri* associated maturation of autophagy (Ogawa et al., 2005; Weddle and Agaisse, 2018). Additionally, septin cages are known to promote autophagy; however, *Shigella* can polymerize actin and avoid septin caging (Krokowski et al., 2018; Lobato-Márquez et al., 2019). Hence, *S. flexneri* has developed an intricate machinery to avoid autophagy and establish infection within the host. As drug resistance is a major problem to tackle *S. flexneri,* thus boosting autophagy can curb intracellular *S. flexneri* infection.

Autophagy induction occurs by inducing several signaling molecules like mTOR, Akt, and AMPK (Floto et al., 2007; Rubinsztein et al., 2012; Young et al., 2020). Recent studies have shown the importance of a transcription factor known as TFEB (Ammanathan et al., 2020). TFEB activation leads to upregulation of autophagy genes. Boosting gene transcription induces host defense machinery and helps in the eradication of pathogens. Therefore, we searched for potent inducers of autophagy.

Capsaicin, a dietary compound from chilli plants, is reported to have anticancer properties (Lin S. R. et al., 2017). Emerging evidences have pointed out that capsaicin also possess antimicrobial activities against gastrointestinal pathogens (Füchtbauer et al., 2021). However, to date, there are no reports of its antibacterial action against *Shigella flexneri*.

Herein, we exploited the mechanism of autophagy induced by capsaicin to inhibit bacterial growth. This study showed for the first time that Caps induced an autophagy-dependent clearance of intracellular *S. flexneri*. Our findings indicate that Caps is a potential autophagy inducer that helps reduce *S. flexneri* infection.

## 2 Materials and Methods

### 2.1 Bacterial Culture Conditions

*Shigella flexneri* (sf2457T) serotype 2a, plasmid-cured *Shigella flexneri* (sf2457T)2a, and *Shigella flexneri*-resistant strain (NA/CIP/NOR/OFX/TET/S/C/AM/E/ST) (BCH12702) were obtained from Dr Asish Mukherjee and Dr. Hemanta Koley (NICED, Kolkata, India). Bacteria were routinely cultured in Mueller Hinton Broth (Himedia) at 37°C incubator with a shaker.

### 2.2 Cell Culture Conditions

HT-29 (ATCC HTB-38) and murine macrophage RAW264.7 (ATCC TIB-71) cells were used during this study. HT-29 cells were cultured with a McCoy’s 5A medium (Sigma-Aldrich) containing 10% heat inactivated FBS (Sigma-Aldrich, United States), 1% non-essential amino acids, and 1% penicillin–streptomycin (Himedia, India). Graphs represent CFU/ml of intracellular *S. flexneri*. RAW264.7 cells were grown in RPMI1640 with 10% FBS and 1% penstrep. Experiments were conducted in cells with maximum three to five passages. Cells were maintained in a 37 °C humidified incubator with 5% CO_2_.

### 2.3 Plasmids

The eGFP-LC3 plasmid was a gift from Prof. Parimal Karmakar (Department of Biotechnology, Jadavpur University, India). pEGFP-N1-TFEB plasmid was a gift from Dr. Ravi Manjithaya, Autophagy lab, JNCASR, Bangalore. Control EGFP plasmid was a kind gift from Dr Santa Sabuj Das, ICMR, NICED, Kolkata. The plasmids were transformed in DH5α and purified by the mini-prep plasmid isolation kit (Promega). The plasmid DNA was suspended in nuclease free water and stored at −20°C.

### 2.4 MTT Assay

Cells were seeded in 96-well plates (1×10^4^ cells/ml). At this seeding density, cells were treated with varying concentrations of Caps (16 μM, 32 µM) keeping an uninfected DMSO control and incubated for 24 h. Cell viability was checked using a Colorimetric Cell Viability Kit IV (MTT) (Promokine) followed by the addition of 20 μl of MTT reagent and incubated for 4 h at 37°C in a CO_2_ incubator. Thereafter, 300 μl of DMSO was added to solubilize the purple crystal formazan, OD was measured at 570 nm, and % viability was calculated and graphically represented.

### 2.5 GFP-LC3B Fluorescence Assay and anti-*Shigella* Immunofluorescence

GFP-LC3 (2 ug/well) plasmid was transfected in HT 29 and Raw264.7 cells using lipofectamine 2000 reagent (Invitrogen. United States). At 85% confluency, cells were transfected with [plasmid (ug): lipofectamine (ul) = 1:3] following the manufacturer’s protocol. After transfection for 48 h, cells were pre-treated with caps (16 µM) followed by *S. flexneri* infection. For *Shigella* immunofluorescence, GFP-LC3B-transfected cells were infected with *S. flexneri*. 400 µl of *S. flexneri* from an overnight culture of O.D (0.5–0.6) was added in intestinal cells and macrophages. Cells were further subjected to immunofluorescence. Briefly, fixed cells were incubated with anti-Shigella antibody (1:250) (ab65282) dilution. Subsequently, TRITC-conjugated anti rabbit secondary antibody (1:2000) (Cat# AP132R) was used. Finally, coverslips were mounted on a ProLong™ Gold Antifade reagent containing DAPI (ThermoFisher) and observed under an inverted confocal microscope (Carl Zeiss LSM 710). GFP-positive cells per sample were selected for observation.

### 2.6 siRNA Transfection

The following siRNAs were purchased from IDT and used for the knockdown of those particular genes: ATG5 siRNA (Design ID hs. Ri. ATG5.13.1) and TFEB siRNA (Design ID hs. Ri. TFEB.13.1). HT-29 cells at 60%–70% confluency were transfected with 12 pmol of siRNA/Scrambled siRNA, and 2 ul of lipofectamine was added per well in a 6-well plate. Forty-eight hours after transfection, the infection assay was performed in cells infected with *S. flexneri* with and without Caps pre-treatment. Cells were lysed for CFU/ml counting.

### 2.7 Immunofluorescence Microscopy

For fluorescence microscopy, cells were grown on coverslips and Caps treatment was done for 2 h. After fixation with 4% formaldehyde, cells were further blocked in PBS containing 3% BSA and 0.1% Triton X100 for 1 h at room temperature. Overnight incubation was performed with anti-TFEB antibody (1:250 dilution). Following that, fixed cells were kept in a blocking buffer containing TRITC-conjugated anti-rabbit secondary antibody (1:2000) (Cat# AP132R). Finally, the coverslips mounting upon glass slides were done by adding a drop of ProLong™ Gold Antifade reagent containing DAPI (ThermoFisher). Images were scanned on a Zeiss LSM 710 confocal system (Carl Zeiss).

### 2.8 Infection Assay

Intestinal and macrophage cells (1X10^5^) were cultured in 6-well plates for 24 h at 37 °C and 5% CO_2_ prior to infection. Cells were further incubated overnight in antibiotic free media. *S. flexneri 2457t* colonies (red) were picked from Congo red plates as Congo red indicates the retention of virulence plasmid. *S. flexneri* was grown overnight in MHB at 37°C with shaking and sub-cultured to an OD_600_ of 0.5–0.6. Bacteria pellets were collected by centrifuging the culture at 12,000 g for 5 min at 4°C and further washed with PBS. Pellets were then re-suspended in incomplete tissue culture media. Intestinal cells were pre-treated with Caps (1.6, 3.2, 4.8, 6.4, 8, 16, 32 µM, or vehicle control (DMSO), for 2, 4, 6, 8, 12, and 24 h in incomplete media. Then, infection with *S. flexneri* was performed at an MOI of 200:1. The infected plates were centrifuged for 15 min at 700 g for synchronization and incubated for 2 h to allow bacteria to enter the cells. Cells were thoroughly washed and treated with 50 μg/ml of gentamicin for 2 h, killing the extracellular bacteria. Further washing with PBS was done, and then cells were lysed using 0.1% triton X. The cell lysates were plated in MHA at 37°C for 18 h to count the number of colonies (CFU/ml). For post-treatment analysis, cells were infected with bacteria and then exposed to Caps for different time points. After incubation and infection, gentamicin was used to kill the extracellular bacteria, and further lysis was done and plated. This assay was also performed using *S. flexneri* highly resistant strain (BCH12702) with or without Caps 12 h post-treatment to compare invasion. Results were compared to 3-methyladenine (#SAE0107, sigma)-treated cells. For infection in macrophages, cells were preincubated with Caps for 2 h followed by 30 min of *S. flexneri* infection (MOI100). After infection, gentamicin was used for killing the extracellular bacteria and further lysis was done and plated. For post-infection analysis, cells were infected with *S flexneri* (MOI100) for 30 min followed by Caps treatment for 4 h. After that, gentamicin was used to kill the extracellular bacteria, and further lysis was performed and plated.

siATG5, siTFEB-transfected cells were pre-treated with Caps and infected for gentamicin survival assay to count CFU/ml compared to scrambled siRNA. TFEB overexpression was also performed by lipofectamine 2000 using pEGFP-N1-TFEB plasmid and empty vector (pcDNA3-EGFP).

### 2.9 Real-Time PCR

RNA isolation was performed using the RNA isolation kit (zymo research) from treated and untreated cells. TRIzol reagent was used for colon samples collected from mice. RNA samples were converted to c-DNAs using the Thermo Scientific c-DNAs synthesis kit. Real-Time PCR was done using SYBR green kit of Applied Biosystems. The expression patterns were analyzed using the ^ΔΔ^Ct method and normalized using the internal control GAPDH. ^ΔΔ^Ct were calculated by the following formula:

$${}^{\text{ΔΔ}}\text{Ct }=\text{ test }-\text{ internal control}-\text{test control}$$

Human primer sequences used were listed as follows:

**Table:** 

Gene	Forward primer	Reverse primer
GAPDH (human)	5′-GTCTTCACCACCATGGAGAAGGC-3′	5′-CATGCCAGTGAGCTTCCCGTTCA-3′
MAP1LC3B (human)	5'-ACCATGCCGTCGGAGAAG-3'	5'-GGTTGGATGCTGCTCTCGAA-3'
ATG5 (human)	5'-GCAAGCCAGACAGGAAAAAG-3'	5'-GACCTTCAGTGGTCCGGTAA-3'
GAPDH (mouse)	5′-GATCTTCGACAAGGGAGCTAAA-3′	5′- TCGCATTCTTCTACACGATAACA-3′
MAP1LC3B (mouse)	5-GTCCTGGACAAGACCAAGTTCC-3′	5′-CCATTCACCAGGAGGAAGAAGG-3′

Differential expression of genes involved in autophagy pathway was analyzed using a human autophagy PCR array. This array contained 84 key genes in a 96-well plate format. Fold change was plotted in a column graph to observe the differences among uninfected, infected, and infected with Caps. PCR array (#PAHS-084Z) (RT^2^ Profiler™ PCR Array Human Autophagy) was performed to understand the complete autophagy profile of the control and treated samples following the manufacturer's instructions (Qiagen). RNA isolation was performed from two groups of cells. cDNA was obtained using cDNA synthesis reagents and mixed with SYBR Green Mastermix (provided inside the kit). Quantitative real-time PCR was done in Applied biosystems StepOnePlus system. Obtained melt curves and ^ΔΔ^Ct values were analyzed in free web-based RT^2^ Profiler™ PCR array data analysis software.

### 2.10 Western blot analysis

Untreated, treated, and infected cell lysates were prepared in RIPA (radioimmunoprecipitation assay buffer) lysis buffer (10 mM Tris–HCl, pH 8.0, 1 mM EDTA, 0.5 mM EGTA, 1% Triton X-100, 0.1% sodium deoxycholate, 0.1% SDS, 140 mM NaCl, 1 mM PMSF) (protease and phosphatase inhibitor added). Lysates were centrifuged at 7,000 rpm for 20 min at 4°C. Nuclear and cytosolic extracts were prepared by using NE-PER™ Nuclear and Cytoplasmic Extraction Reagents of Thermo Scientific (Cat#78833) following the manufacturer’s instructions.

After protein estimation, lysates were boiled in SDS-PAGE sample buffer and run on 10% or 12.5% SDS- PAGE gel at 120 V. Gels were transferred to PVDF membrane. Blocking was performed with 5% skimmed milk dissolved in TBST (20 mM Tris–HCl, 150 mM NaCl, 0.1% Tween20) buffer for 1 h at room temperature, and the membranes were incubated overnight with primary antibodies at 4 °C. The primary antibodies utilized are rabbit monoclonal anti-GAPDH antibody (Cat#D16H11) (1:2,000), rabbit polyclonal Anti-SQSTM1/P62 antibody (Cat# ab91526) (1:1,000), rabbit monoclonal anti-ATG5 (Cat #ab228668) (1:1,000), rabbit monoclonal anti-beclin1  antibody (Cat #ab207612) (1:1,000), rabbit polyclonal anti-LC3B antibody (Cat #ab51520) (1:1,000), and rabbit monoclonal anti-TFEB antibody (Cat #D2070). Following primary antibody treatment, membranes were kept in a shaker at room temperature for 2 h with horseradish peroxidase (HRP) conjugated goat anti-rabbit secondary antibody (dilution 1:10,000) or goat anti-mouse secondary antibody (dilution 1:10,000). Membranes were finally washed with TBST for 30 min. Bands were observed in the ChemiDoc MP Imaging System (Biorad) using Millipore immobilon western chemiluminescent HRP substrate (luminol and hydrogen peroxide) as a substrate.

### 2.11 Broth Dilution Assay

A broth dilution assay was performed in the presence of DMSO as a control, 16, 32, 64, and 80 µM Caps, and nalidixic acid (10 μg/ml) as a positive control in separate flasks containing MHB. A total of 10^5^ *S. flexneri* from overnight culture was added to the flasks. O.D._600nm_ was measured at regular intervals and graphically represented.

### 2.12 Determination of the Minimum Inhibitory Concentration

*Shigella flexneri* (BCH12702) was treated with antibiotics (NA/CIP/NOR/OFX/TET/S/C/AM/E/ST) at different concentrations. The minimum inhibitory concentrations of the antibiotics were determined by the CLSI broth microdilution method. The test was performed by serially diluting the antibiotics in LB media and incubating for 24 h in a 37°C incubator. The minimum inhibitory concentration (MIC) represents the lowest concentration of the antibiotics that resulted in a lack of visible bacterial growth. Tabular representation shows the MIC (µg/ml) and cutoff values for resistance according to CLSI guidelines.

### 2.13 Electron Microscopy

Uninfected and infected cells (HT-29 and RAW264.7) treated with or without Caps were fixed in 3% glutaraldehyde in 0.1 M sodium cacodylate buffer. Further fixation with 1% osmium tetroxide continued, and then dehydrated with increasing grades of acetone, eventually embedded in Agar 100 resin, and polymerized at 60 °C. Ultrathin sections (around 40–50 nm thickness) were made using a Leica Ultracut UCT ultramicrotome (Leica Microsystems, Germany), picked up on nickel grids, and dual-stained with 2% aqueous uranyl acetate and 0.2% lead citrate. We examined cell sections using an FEI Tecnai 12 Biotwin transmission electron microscope (FEI, Hillsboro, OR, United States) at an accelerating voltage of 100 kV. Percentage autophagosome formation was calculated: Cell number containing autophagosome/total cell number x 100%Autophagic bodies were outlined with the help of Adobe Photoshop.

### 2.14 Chromatin Immunoprecipitation Assay

HT-29 cells were treated with Caps for 4 h. After incubation, crosslinking was done in 1% formaldehyde and processed according to the protocol of the CHIP assay kit (#7-295; Merck Millipore). Briefly, cells were lysed in SDS lysis buffer and pelleted. After processing, chromatin was immunoprecipitated with anti-TFEB antibodies (5 µg/sample). The chromatin fraction, which lacks a primary antibody, was taken as “input.” Real-time PCR was performed using the following chip primer assemblies. Primers were designed for amplifying CLEAR elements in the promoter region of TFEB using primer: MAP1LC3B-forward; 5′-GAA​GGC​TCG​GGA​CAA​AAG​CAG-3′, reverse; 5′-GTG​GGT​GGC​TTC​CGG​GGA​G-3’. The PCR cycle was conducted in accordance with the manufacturer’s instructions. The data were graphically represented as the % of input.

### 2.15 Transcription Factor Assay

The TFEB transcription factor assay was performed using the RayBio^®^ Human TFEB TF-Activity Assay Kit Protocol. Nuclear extract collected from control and Caps-treated cells were used as samples. Samples were added to wells coated with oligonucleotides containing CLEAR sequence. After the addition of primary antibody, HRP-conjugated secondary antibody was added. This step was followed by the addition of the TMB substrate reagent, the reaction was stopped with stop solution, and O.D. was collected at 450 nm by a spectrophotometer. The fold change was calculated and graphically represented.

### 2.16 *In Vivo* Experimental Design

#### 2.16.1 Animals

Male BALB/c (8 weeks) mice weighing 20–22 g mice were used for *S. flexneri* infection. Animals were adjusted under standard laboratory conditions. Mice were provided with a standard diet and water ad libitum. IAEC (Institutional Animal Ethical Committee), NICED, Kolkata (PRO/157/-July 2022) guidelines were followed during all the experiments. Animals were maintained in the animal house with 75% humidity, and specific pathogen-free healthy individuals were selected.

After fasting for 6 h, mice were injected with pathogenic *Shigella flexneri* (sf2457T) intraperitoneally (Yang et al., 2014). After 2 h, Caps treatment was performed. In the experimental design, four groups (n = 4) were created accordingly: Group 1. Control: Only DMSO was used as the vehicle. Group 2. Infected: Received *S. flexneri* suspension (0.5 × 10^9^ CFU/ml) in PBS. Group 3. Infected + Caps: Received *S. flexneri* suspension followed by Caps (20 mg kg−1 body weight) treatment for 2 h. Group 4. Caps: Received Caps (20 mg kg−1 body weight). Each group was kept in an individual cage. Experiments were repeated in triplicate (n = 3). The total number of mice in each group was 12.

#### 2.16.2 Collection of Colon

After 2 h of Caps treatment, all the animals were sacrificed. Colon from drug-treated, untreated, infected, and control mice was aseptically removed. The crushed colon was plated for bacterial colony count. The tissues were kept at −80 °C until further experiments were performed.

#### 2.16.3 Preparation of Colon Tissue Homogenate

Colon samples were collected following dissection, washed in PBS buffer, and further homogenized in an ice-cold RIPA lysis buffer for western blot and in TRIzol reagent (RNAiso Plus, TaKaRa) for RNA isolation. The homogenates were centrifuged at 12,000 rpm at 4 °C for 10 min. The supernatants collected were further utilized for experiments. RNA was isolated, and RNA to cDNA conversion was performed using a Verso cDNA Synthesis Kit (Thermo Scientific) followed by qRT PCR.

### 2.17 Statistical Analysis

Test information is presented as mean ± S.E.M. Statistical analyses were performed, and bar graphs were processed in GraphPad Prism 5 after the data processing in Microsoft Excel. For comparison between two groups, an unpaired *t*-test was performed, and for multiple comparison, one-way ANOVA is performed. For multiple variants, two-way ANOVA was performed. Significance level has been marked as * for *p* < 0.05, which implies significant, ** for *p* < 0.01, which implies very significant, and *** for *p* < 0.001, which implies highly significant.

## 3 Results

### 3.1 Caps Inhibits Intracellular *S. flexneri* Growth

**FIGURE 1:**
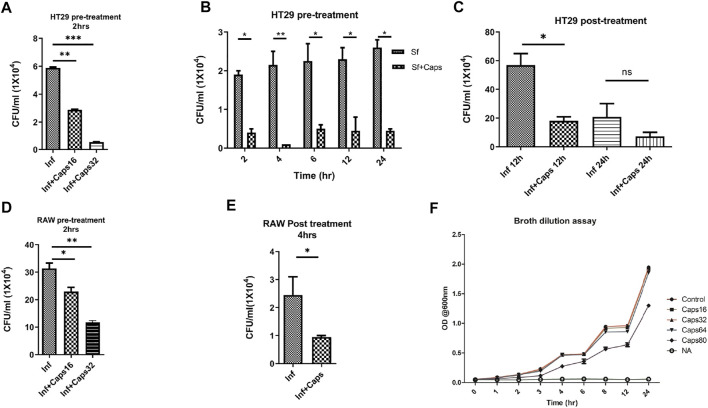
Intracellular *S. flexneri* growth was inhibited by capsaicin (Caps) in both intestinal and macrophage cells. **(A)** Infection assay was performed in HT29 cells with different concentrations of capsaicin (Caps). Briefly, cells were pre-treated (16 µM, 32 µM) with Caps for 2 h followed by *S. flexneri* infection (MOI 200) for 2 h. Finally, gentamicin treatment was done to kill extracellular bacteria. Cells were lysed and plated overnight for counting the number of colonies (CFU/ml). Graphs represent CFU/ml of intracellular *S. flexneri*. One-way ANOVA was performed. **(B)** Caps pre-treatment (16 µM) for 2 h followed by *S. flexneri* (MOI 200) infection at different time points (2, 4, 6, 8, 12, 24 h) was done. Two-way ANOVA was performed. **(C)** HT29 cells were infected with MOI200 followed by post-treatment with Caps (16 µM) for 12 and 24 h. One-way ANOVA was performed. Cells were lysed and plated to count the number of colonies. **(D)** RAW 264.7 cells were infected with *S. flexneri* MOI 100 for 30 min after 2 h Caps pre-treatment. Cells were lysed and plated to count the number of colonies One-way ANOVA was performed. **(E)** RAW264.7 cells were infected with *S. flexneri* MOI 100, followed by post-treatment with Caps (16 µM) for 4 h. Graph represents CFU/ml of intracellular *S. flexneri*. One-way ANOVA was performed. **(F)** A broth dilution assay was performed in the presence of DMSO control, 16, 32, 64, and 80 µM Caps and nalidixic acid (10 μg/ml) (NA) followed by the addition of 10^5^ *S. flexneri* overnight culture. The OD at 600 nm was measured at regular intervals. Graphs were represented using GraphPad Prism 5 as mean ± SEM (n = 3); Significance was calculated; *p < 0.05, **p < 0.01, ***p < 0.001.

The antibacterial effects of capsaicin (Caps) are reported against *Vibrio cholerae*, *H. pylori* and other bacteria (Füchtbauer et al., 2021). However, there are no such reports of capsaicin-induced inhibition of intracellular *S. flexneri* growth. Here, we tested the effect of Caps on the intracellular clearance of *S. flexneri*. For this approach, we used an intracellular invasion assay in an *S. flexneri* infection model. Briefly, Caps was added in HT-29 cells for 2 h followed by *S. flexneri* infection to check intracellular bacterial clearance. Caps at different doses (16 and 32 μM) significantly reduced *S. flexneri* replication (Figure 1A). In addition, Caps significantly (16 μM) reduced *S. flexneri* multiplication after pre-treatment at different time points (2, 4, 6, 12, and 24 h) in HT-29 cells (Figure 1B). Moreover, we checked the effect of Caps by posttreatment analysis. *S. flexneri* growth was inhibited significantly at 12 h by Caps. However, at 24 h, cell death occurred in infected cells; hence, data are not significant (Figure 1C).

Similarly, we validated the results in macrophages, as macrophages are the immune cells and site of infection for *S. flexneri* during pathogenesis. Caps pre-treatment for 2 h significantly reduced bacterial growth in a dose-dependent manner (Figure 1D). Moreover, posttreatment analysis in macrophages showed that Caps is effective at 4 h in inhibiting *S. flexneri* growth (Figure 1E). Upon compound addition, bacterial growth was significantly reduced. However, the inhibition of bacterial burden could be due to a probable antibacterial property of Caps. Hence, we checked the effect of Caps on *S. flexneri* by the broth dilution method for extracellular bactericidal properties. There is no apparent change in bacterial growth for treated (Caps 16, 32, 64, and 80 μM) and untreated samples (Figure 1F). Collectively, the data revealed that exposure to Caps inhibits intracellular *S. flexneri* proliferation.

### 3.2 Capsaicin (Caps) Induces Autophagy in Intestinal Cells

**FIGURE 2:**
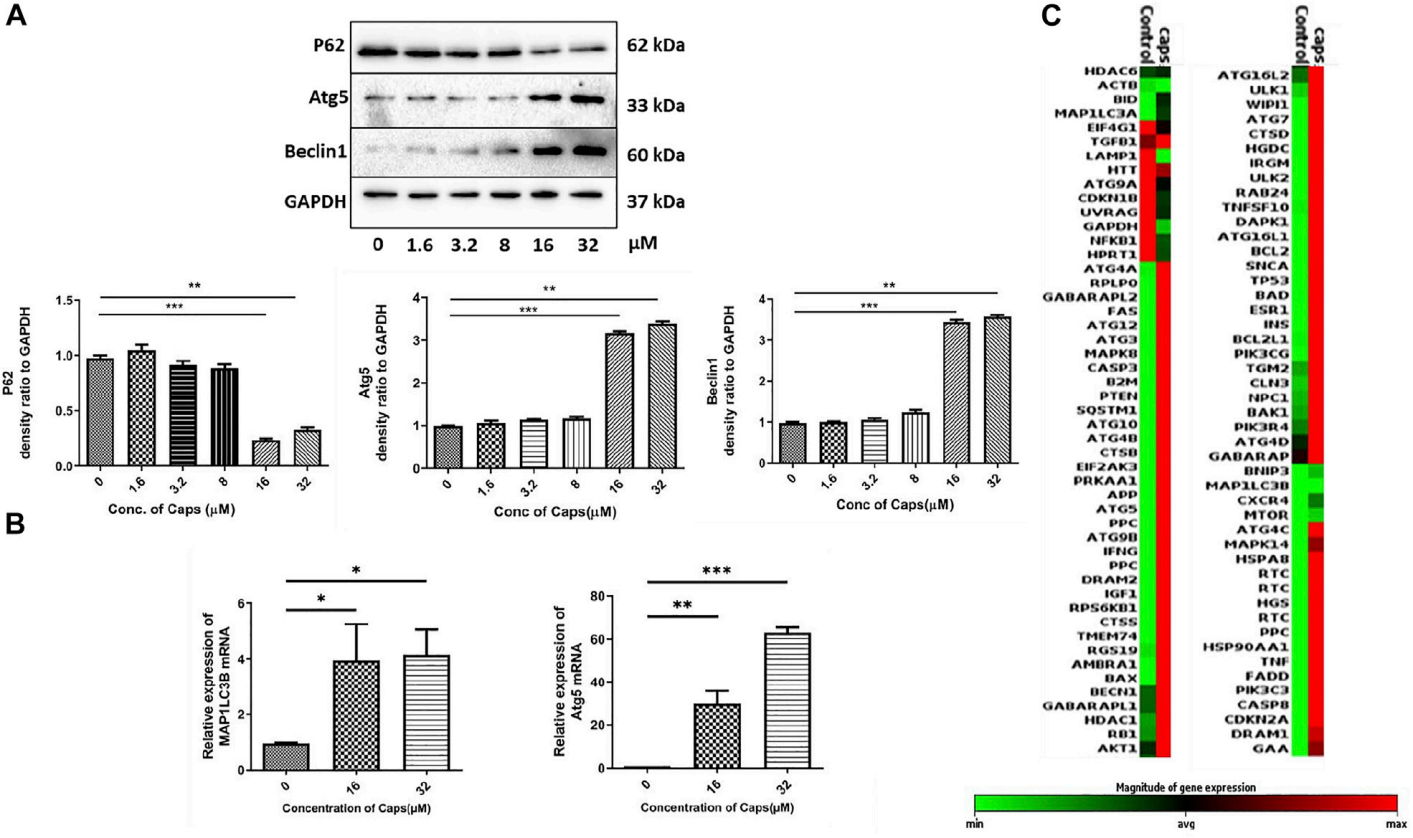
Capsaicin (Caps) induces autophagy in intestinal cells. **(A)** HT29 cells were treated with Caps (1.6, 3.2, 8, 16, 32 µM) or DMSO for 2 h. Expression of major autophagy-associated marker proteins P62, Atg5, and Beclin1 was observed in western blot. GAPDH was used as a loading control. Densitometric analyses are graphically represented. **(B)** HT29 cells were treated with Caps (16, 32 µM) or DMSO for 2 h followed by qRT-PCR, Expression of the autophagy-associated marker genes (Map1LC3B, Atg5) was quantified using GAPDH as a housekeeping gene. One-way ANOVA was performed. **(C)** PCR array was performed with control and only Caps-treated samples. Caps treatment was done for 4 h. Heat map generated represents differential gene expression. All experiments were performed in triplicate. Graphs were generated using GraphPad Prism 5 were represented as mean ± SEM (n = 3); significance was calculated; *p < 0.05, **p < 0.01, ***p < 0.001.

As capsaicin has no direct antibacterial action against *S. flexneri,* we questioned the probable mechanism involved in reducing intracellular bacterial growth. Previously, it was identified that Caps overexpressed autophagy proteins in nasopharyngeal cancer cells (Lin S. R. et al., 2017). However, there are no such reports on intestinal cells. Here, we examined the expression of autophagy proteins in intestinal epithelial cells by western blots. Caps overexpressed the autophagy proteins Atg5 and beclin1 in a dose-dependent manner in HT29 cells after treatment for 2 h (Figure 2A). Beclin1 is an autophagy regulator involved in autophagosome formation, while Atg5 helps in autophagosome maturation and LC3B lipidation (Brier et al., 2019). P62 degradation, another marker of autophagy initiation, takes place due to Caps treatment. Next, we examined the effect of Caps on autophagy genes at different concentrations. Caps significantly augmented the expression of MAP1LC3B and Atg5 genes in a dose-dependent manner (Figure 2B). To further assess the effect of Caps on autophagic gene expression, a PCR array was performed. Differential gene expression was observed due to Caps treatment in intestinal cells for 4 h. More than 78% of the genes are augmented by Caps alone at a dose of 16 µM (Figure 2C). As autophagy induction is often linked with cellular toxicity, we checked the toxicity of Caps in both intestinal and macrophage cells. Cytotoxicity assay of Caps in intestinal and macrophage cells showed insignificant effects due to Caps treatment for 24 h (Supplementary Figure S1). Together, these results indicate that Caps induces autophagic gene and protein expression in intestinal cells.

### 3.3 Treatment With Caps Enhances Autophagosome Formation

**FIGURE 3:**
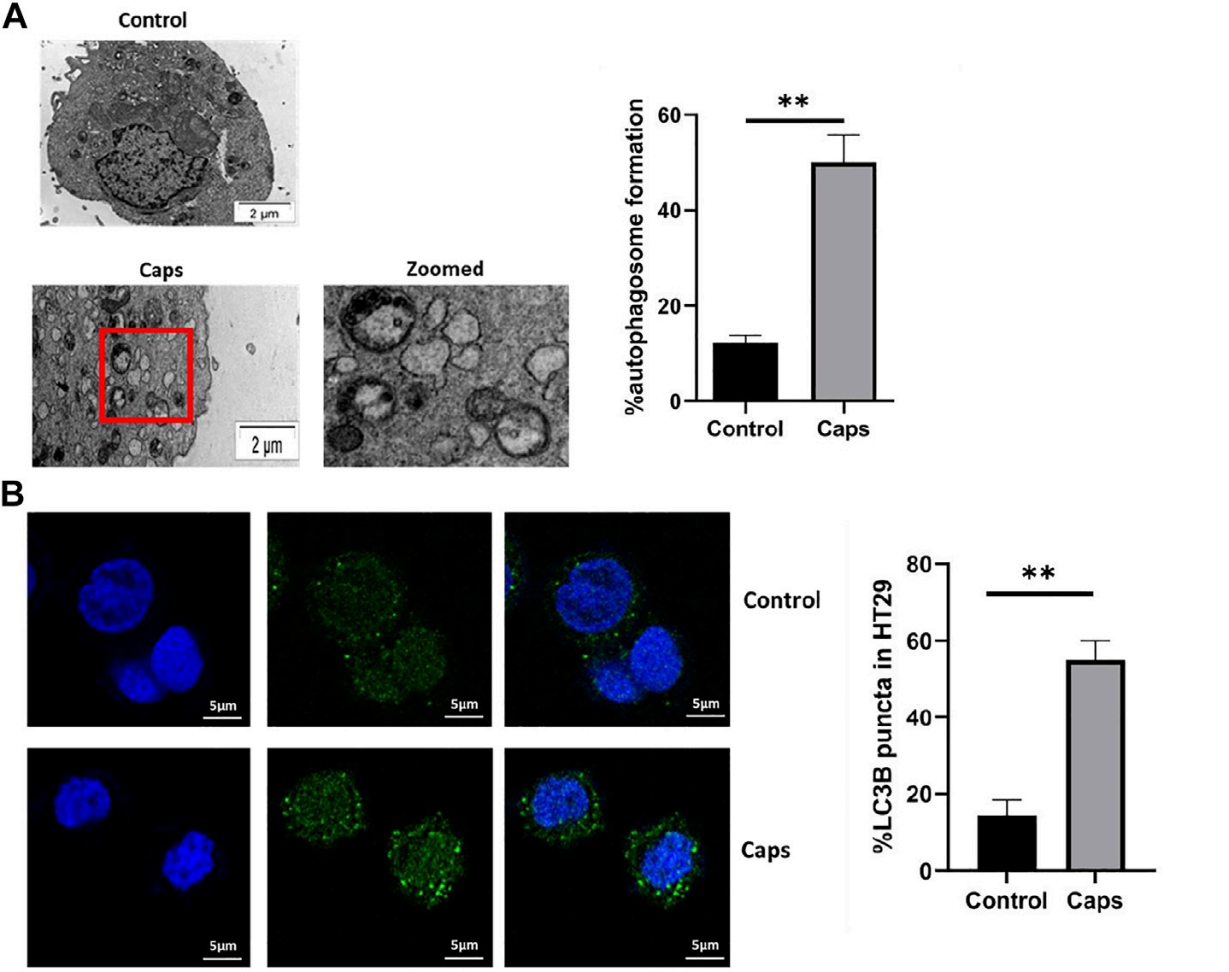
Treatment with Caps enhances autophagosome formation. **(A)** Electron microscopic (TEM) pictures showed the presence of autophagosome formation in control and Caps-treated HT-29 cells for 4 h. Zoomed images of the marked area. Scale bar: 2 μm. Graphical representation of % autophagosome formation as observed in TEM. Unpaired *t*-test was performed. **(B)** Confocal microscopic images of GFP-LC3B transfected intestinal cells (HT-29) showed LC3B puncta (green) formation. Briefly, cells were transfected with GFP-LC3B plasmid, then treated with Caps for 4 h, and visualized under a confocal microscope. Graph representing the percentage of LC3B puncta formation. All experiments were performed in triplicate. Unpaired *t*-test was performed. Graphs generated using GraphPad Prism 5 were represented as mean ± SEM (n = 3); significance was calculated; *p < 0.05, **p < 0.01, ***p < 0.001.

In addition to the stimulation of autophagic gene expression by Caps, we examined the potential effect of Caps on autophagosome formation by transmission electron microscopy (TEM) and confocal microscopy. The images showed autophagosome formation in cells treated with Caps (Figure 3A). Caps treatment alone showed 50% autophagosome formation in intestinal cells. Similar observations were validated by confocal microscopy. It is known that autophagosome formation is associated with membrane-bound LC3B. GFP-LC3B-transfected cells were exposed to Caps. Caps (16 µM) treatment for 2 h resulted in LC3B puncta formation in intestinal cells (Figure 3B). Together, these results confirm that Caps induces autophagosome formation in intestinal cells.

### 3.4 Caps-Induced Autophagy Targets *S. flexneri* to Autophagosome

**FIGURE 4:**
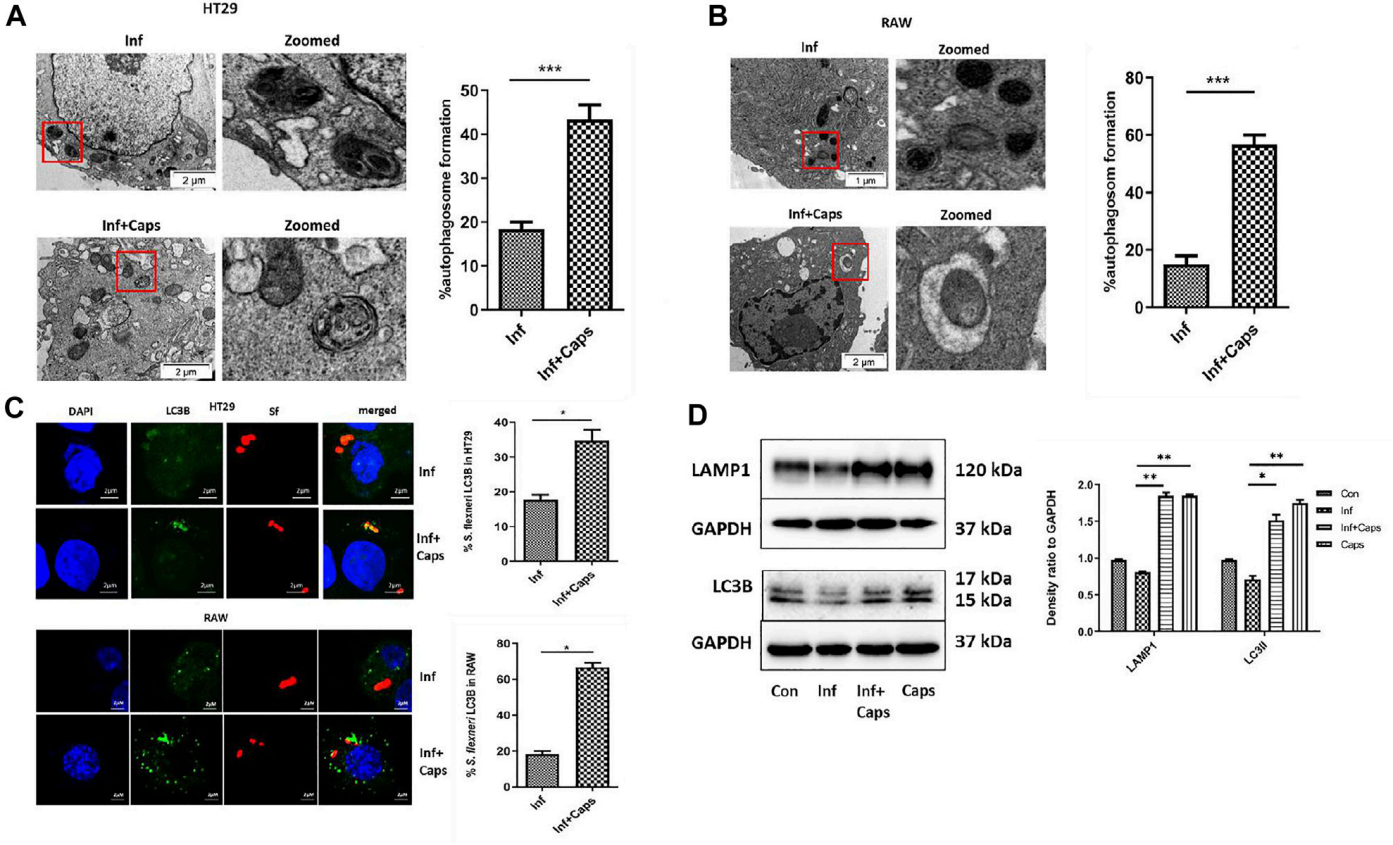
Caps treatment results in the engulfment of bacteria by autophagosome. **(A)** Transmission electron microscopy (TEM) pictures showed the presence of autophagosome formation in infected intestinal cells with and without Caps treatment. Zoomed images of the marked area. Scale bar: 2 μm. Graphical representation of % autophagosome formation as observed in TEM. Unpaired *t*-test was performed. **(B)** Transmission electron microscopy (TEM) pictures of autophagosome formation in infected and infected + Caps RAW267.4 cells. Zoomed images of the marked area. Scale bar: 2 μm. **(C)** Microscopic images of GFP-LC3B transfected intestinal cells (HT-29) showed the recruitment of *S. flexneri* (red) to LC3B (green). Briefly, cells were transfected with GFP-LC3B plasmid, then treated with Caps for 2 h followed by *S. flexneri* infection for 2 h. Graph representing the percentage of bacteria localizing with LC3B. Microscopy images of GFP-LC3B transfected macrophages (RAW 267.4) infected with bacteria and treated with and without Caps. Graph representing the number of bacteria recruited to LC3B. Unpaired *t*-test was performed. Scale bar: 2 μM. **(D)** HT29 cells were pre-treated with Caps for 2 h followed by infection. After infection, cells were lysed. Cell lysates were subjected to immunoblotting. Increase in LC3B and LAMP1 expression was shown by western blotting, and densitometric analyses are graphically represented [Caps = Only Caps treatment at a dose of 16 µM]. Graphs were represented using GraphPad Prism 5 as mean ± SEM (n = 3); one-way ANOVA was performed. Significance was calculated; *p < 0.05, **p < 0.01, ***p < 0.001.

Host-induced capture of pathogens by autophagosomes occurs during autophagy-mediated degradation. As *S. flexneri* is known to escape from autophagy, we checked the capture of bacteria by autophagosomes due to Caps treatment in infected cells. Caps treatment showed the presence of autophagosome-like structures containing bacteria. We calculated the percentage of autophagosomes and observed that approximately 40% autophagosome formation takes place in *S. flexneri*-infected cells pre-treated with Caps (Figure 4A). Similar observations were observed in macrophage cells. Capture of bacteria by autophagosome was observed in drug-treated cells (Figure 4B). All these findings direct that Caps treatment induced autophagosome formation in *S. flexneri*-infected cells.

To confirm the capture of *S. flexneri* by autophagosomes, confocal microscopy was performed. Therefore, both macrophages and intestinal cells were transfected with GFP-LC3B, treated with Caps (16μM) for 2 h, and finally infected with bacteria. Caps treatment resulted in the increased recruitment of LC3B to bacteria. In intestinal cells, almost 34% of bacteria were localized with LC3B, whereas in macrophages, 60% of bacteria were bound with LC3B puncta, suggesting that autophagy is a tool for Caps-mediated bacterial clearance (Figure 4C). Moreover, the T3SS null strain (plasmid cured) was taken and the effect of Caps was observed. *Shigella flexneri* showed white colonies on Congo red plates instead of red as these colonies are defective in the activation of virulence factors (T3SS) (Supplementary Figure S2). Confocal microscopy revealed that the recruitment of bacteria to LC3B was absent in Caps-treated cells. Most of the bacteria were outside the cells. In addition, we verified Caps-induced activation of autophagy by western blot in infected and uninfected cells. Both LC3B and LAMP1 proteins, the end stage markers of autophagy, were induced in drug-treated (both infected and uninfected) cells (Figure 4D). Overall, these results suggest that Caps-induced autophagy is responsible for targeting bacteria to the autophagosomes.

### 3.5 Inhibition of Autophagy Increases *S. flexneri* Growth

**FIGURE 5:**
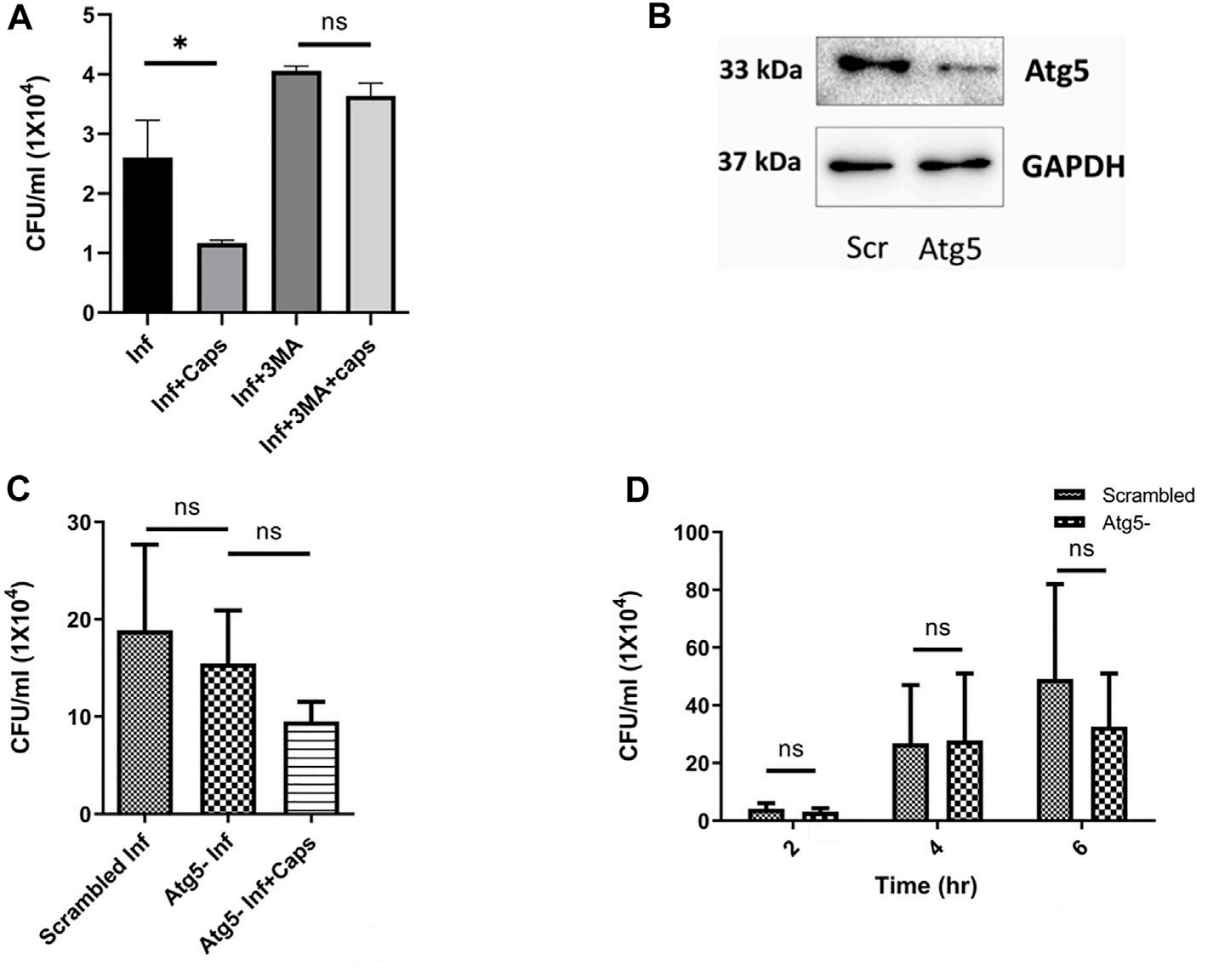
Autophagy inhibition induced bacterial growth. **(A)** Intestinal cells were treated with 3MA (5 mM), Caps (16µM) for 2 h, further infected with *S. flexneri* for 2 h followed by gentamicin treatment. Infection assay was performed. Graph represents intracellular bacterial count. One-way ANOVA was performed. **(B)** Cells were transfected with scrambled and Atg5 siRNA. Western blot shows the Atg5 expression in scrambled and knockdown cells. GAPDH was kept as a housekeeping control. **(C)** Infection assay was performed in scrambled and Atg5 knockdown cells treated with Caps for 2 h followed by infection. CFU/ml was graphically represented. One-way ANOVA was performed. **(D)** Infection assay was performed in scrambled and Atg5 knockdown cells for different time points (2, 4, 6 h). Briefly, after transfection with scrambled and Atg5 siRNA, cells were infected for different time points. Cells were lysed and plated for CFU count. Graphs were represented using GraphPad Prism 5 as mean ± SEM (n = 3); significance was calculated by two-way ANOVA; *p < 0.05, **p < 0.01, ***p < 0.001.

Following our investigations, we tried to find out the impact of autophagy inhibition on *S. flexneri* growth. We used autophagy inhibitors like 3-methyl adenine (3MA). As expected, 3MA increased intracellular *S. flexneri* proliferation. In the presence of 3MA, the anti-shigella activity of Caps was impaired, suggesting that the killing of bacteria is dependent on autophagy (Figure 5A). To rule out the nonspecific effect of inhibitors (3MA) a major autophagy player Atg5 was knocked down using siRNA to inhibit autophagy. Successful Atg5 knockdown was confirmed by western blot (Figure 5B). In Atg5 knockdown cells, Caps treatment failed to inhibit *S. flexneri* intracellular growth (Figure 5C).

Furthermore, to observe the effect of Atg5 knockdown in detail, we infected Atg5 knockdown cells for different time intervals (Figure 5D). Bacterial growth was unaffected due to the blockade of autophagy. These observations indicate that Caps-induced autophagy is responsible for *S. flexneri* death.

### 3.6 Capsaicin (Caps) Triggers Autophagy Gene Transcription by Nuclear Localization of TFEB

**FIGURE 6:**
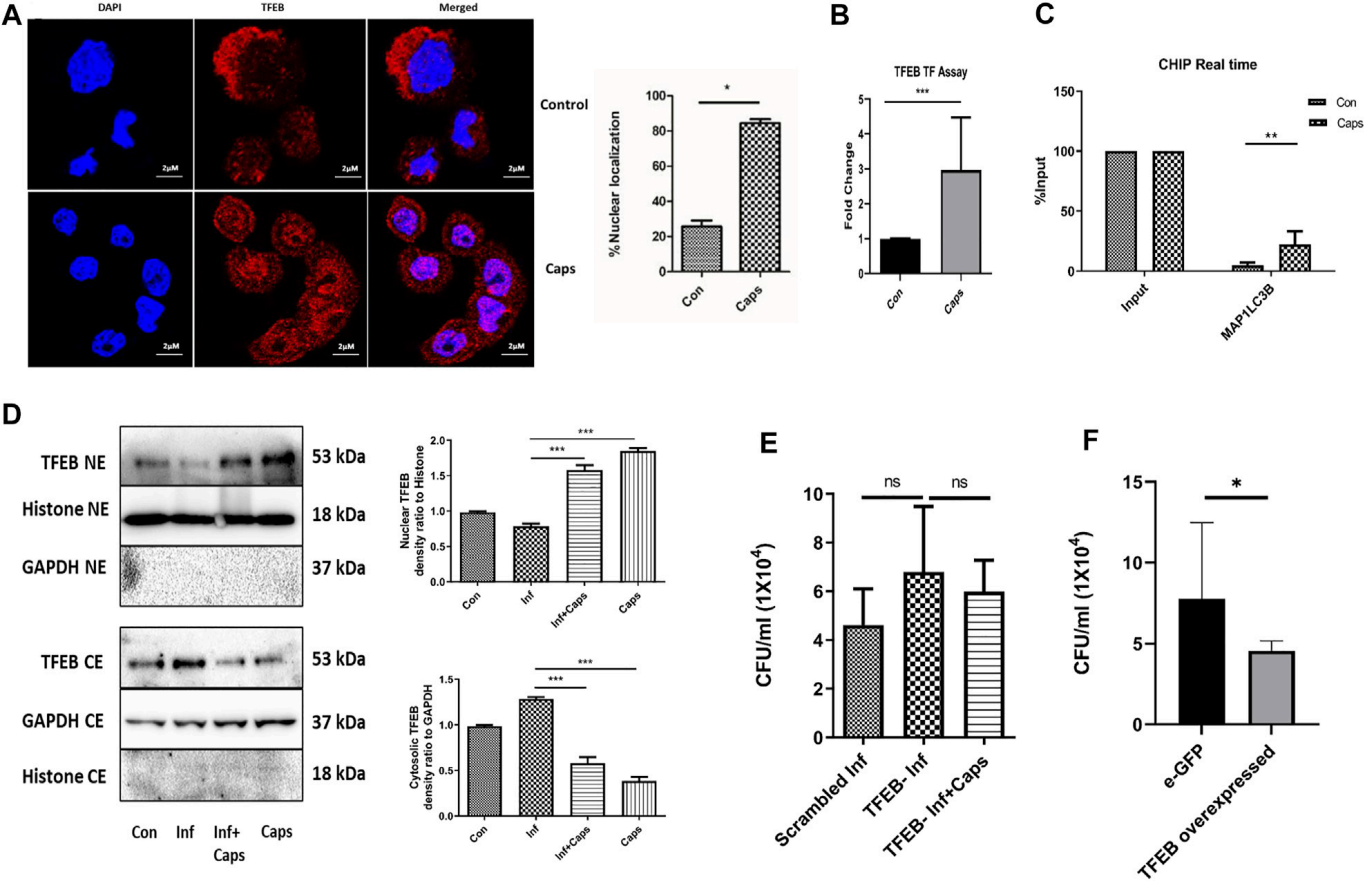
Caps treatment induced TFEB nuclear localization. **(A)** Confocal microscopic data showing nuclear localization of TFEB upon Caps treatment. Briefly, HT29 cells were treated with Caps (16 μM) for 2 h followed by immunofluorescence microscopy. Scale bar: 2 μm. **(B)** Caps treated and untreated cells were subjected to TFEB transcription assay. Transcription factor assay was performed to show the activation of TFEB binding to CLEAR sequence by Caps. Unpaired *t*-test was performed. **(C)** CHIP assay was performed with or without anti-TFEB antibody (input). Expression of MAP1LC3B was checked and compared to input in real-time PCR assay for untreated and Caps-treated intestinal cells. The data were represented graphically and compared to input; two-way ANOVA was performed. **(D)** Western blot analysis was performed for determining the protein level of TFEB in both cytosolic (CE) and nuclear extracts (NE) after Caps treatment in *S. flexneri* infected and uninfected cells. GAPDH was kept as a housekeeping control for cytoplasmic cell extracts. Histone was used as a control for nuclear extracts. Densitometric analyses are shown and graphically represented. One-way ANOVA was performed. **(E)** Cells were transfected with scrambled siRNA or TFEB siRNA. After transfection, cells were treated with Caps for 2 h and infected with *S. flexneri* for 2 h followed by gentamicin treatment. Cell lysis was performed and plated. One-way ANOVA was performed. **(F)** Cells were transfected with an empty vector eGFP or eGFP-TFEB plasmid followed by *S. flexneri* infection. Cells were lysed and plated for colony count. Unpaired *t*-test was performed. Graphs were represented using GraphPad Prism 5 as mean ± SEM (n = 3); Significance was calculated; *p < 0.05, **p < 0.01, ***p < 0.001.

As Caps induced the transcription of autophagy genes, we searched for a potential transcription factor responsible for autophagosomal and lysosomal biogenesis. TFEB, a transcription factor, is reported to enhance autophagosome biogenesis. It binds to the promoter of CLEAR network (coordinated, lysosomal expression, and regulation) and induces autophagy genes (Polito et al., 2014; Sardiello, 2016; Vega-Rubin-de-Celis et al., 2017; Fan et al., 2018; Puertollano et al., 2018). HT29 cells were treated with Caps for 2 h followed by immunofluorescence. Herein, we observed an 80% increase in the nuclear expression of TFEB in Caps-treated cells as compared to that of untreated cells by the immunofluorescence assay (Figure 6A). To confirm TFEB nuclear localization and autophagic gene transcription, we performed promoter assays. Consistently, Caps augmented TFEB promoter activity significantly in treated cells as compared to that of untreated cells by binding to CLEAR sequence (Figure 6B). ChIP assay after chromatin immunoprecipitation also confirmed higher TFEB binding to MAP1LC3B promoter in cells treated with Caps (Figure 6C). Therefore, Caps triggered autophagic gene transcription via TFEB. Moreover, we examined the effect of Caps by immunoblotting. Caps treatment enhanced the nuclear expression of TFEB in both *S. flexneri*-infected and uninfected cells, whereas Caps reduced the cytosolic expression of TFEB (Figure 6D). These results suggest that Caps induced autophagic gene transcription by stimulating the nuclear translocation of TFEB.

To evaluate the relevance of TFEB activation in *S. flexneri* proliferation, we silenced TFEB by siRNA. TFEB knockdown itself has no effect on *S. flexneri* growth; however, Caps treatment in TFEB-silenced cells fails to clear intracellular bacterial burden (Figure 6E). Similarly, the overexpression of eGFP-TFEB plasmid in HT 29 cells resulted in the reduction of *S. flexneri* growth significantly (Figure 6F). Silencing of TFEB by siRNA and overexpression in intestinal cells were confirmed by immunoblot analysis (Supplementary Figure S3). Taken together, these findings indicate that TFEB is a major player in reducing bacterial growth by Caps.

### 3.7 Capsaicin (Caps) Inhibits Intracellular Survival of *S. flexneri* in Both Cellular and Animal Models

**FIGURE 7:**
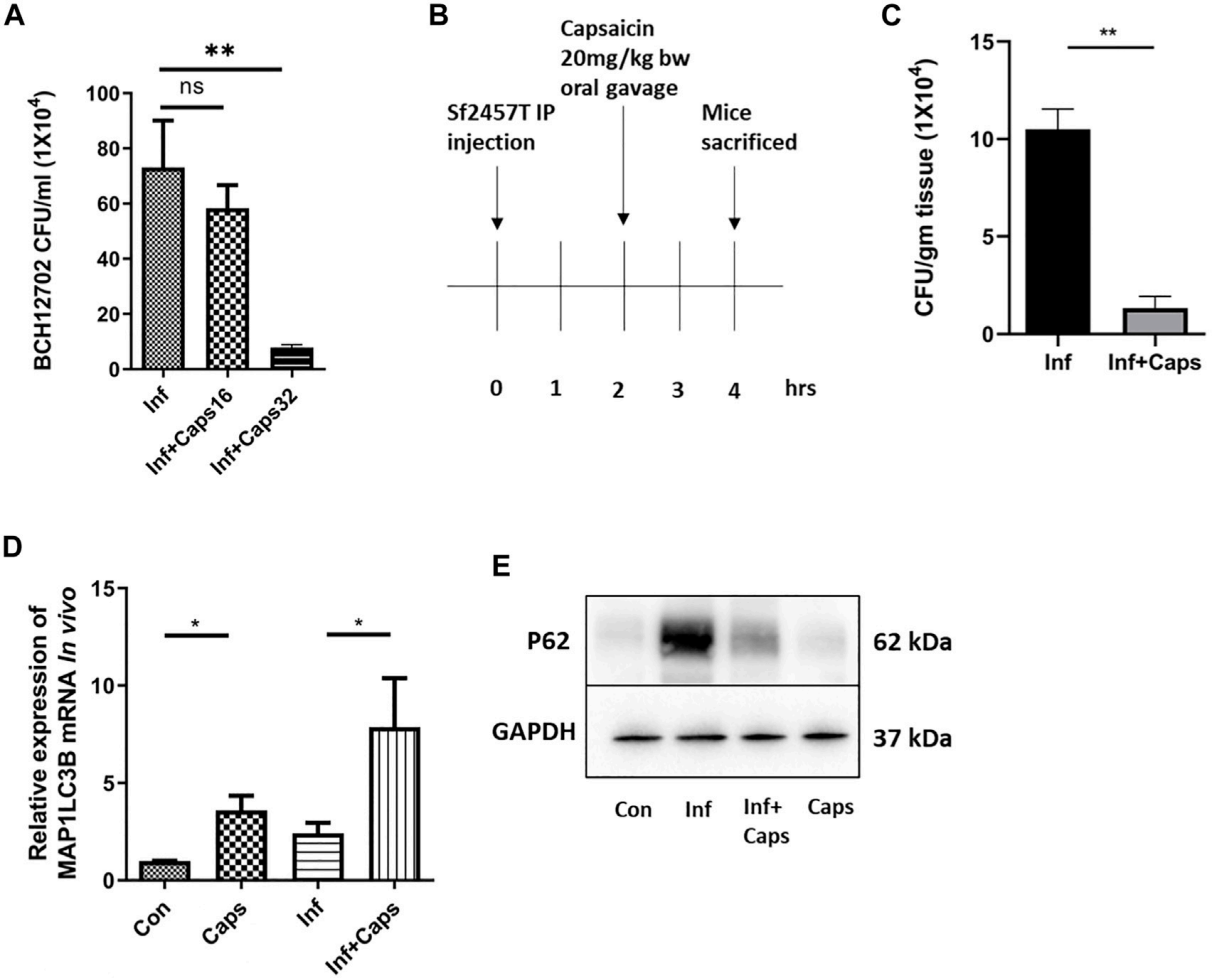
Caps reduced bacterial growth in both cellular and *in vivo* conditions. **(A)** Cells were infected with resistant *S. flexneri 2a* (BCH12702) strain followed by 16 μM, 32 µM Caps post-treatment for 12 h and CFU/ml was graphically represented. One-way ANOVA was performed. **(B)** Caps treatment in *Shigella flexneri 2457T*-infected BALB/c mice. Briefly, mice were infected for 2 h followed by Caps (20 mg/kg b. w.) treatment for 2 h. Four groups (N = 4) were created accordingly: Group 1. Control: only DMSO was used as the vehicle. Group 2. Infected: Received *S. flexneri* suspension (0.5 × 10^9^ CFU/ml) in PBS. Group 3. Infected + Caps: Received *S. flexneri* suspension followed by Caps (20 mg kg^−1^ body weight) treatment for 2 h. Group 4. Caps: Received Caps (20 mg kg−1 body weight). Each group was kept in an individual cage. Experiments were repeated in triplicate (n = 3). Total number of mice in each group were 12. **(C)** CFU was counted and plotted for *S. flexneri sf2457T* infected and infected + Caps-treated crushed colon samples. Unpaired *t*-test was performed. **(D)** qRT-PCR was performed for the expression of the autophagy-associated marker gene MAP1LC3B in control, infected, infected with caps treatment, and only Caps-treated mice tissues using GAPDH as a housekeeping gene control. One-way ANOVA was performed. **(E)** Expression of major autophagy-associated marker protein P62 was observed in western blot. GAPDH was used as a loading control. Graphs were represented using GraphPad Prism 5 as mean ± SEM (n = 3); significance was calculated: *p < 0.05, **p < 0.01, ***p < 0.001.

To assess the efficacy of Caps in controlling *S. flexneri* infection, we checked the effect of Caps against a resistant strain of *S. flexneri*. We observed that Caps is effective in inhibiting the resistant *S. flexneri* strain growth significantly at a higher dose of 32 µM after 12 h post treatment. Supplementary Table S1 shows the MIC values of the resistant strain against different antibiotics (Figure 7A). Finally, we validated the effect of Caps in mouse model. Mice were infected by intraperitoneal challenge with *S. flexneri* 2457T strain (Figure 7B). The dose of Caps was selected in the infection model based on the available data of capsaicin (Rollyson et al., 2014). After 2 h of infection, mice were treated with Caps (20 mg/kg body weight) for 2 h and then sacrificed for further studies. We took the colon from infected and Caps-treated mice samples. The bacterial count showed a decrease in *S. flexneri*-infected mice that received Caps (Figure 7C). Additionally, we examined the effect of Caps on MAP1LC3B gene expression in mice colon tissues. Caps significantly enhanced the MAP1LC3B gene expression in infected mice as compared to that in untreated mice. Caps alone also increased the MAP1LC3B gene expression significantly (Figure 7D). We assessed the effect of Caps on p62 degradation, another marker of autophagy induction. Caps treatment induced p62 degradation in both infected and uninfected mice tissues (Figure 7E). These data confirm that Caps has the potential to be used as a therapeutic against *S. flexneri*.

## 4 Discussion

There is a constant interplay between the host and pathogen to maintain cellular homeostasis. Both play diverse strategies to overcome each other. One of the powerful strategies to boost host defense is autophagy. However, intracellular pathogens evade autophagy to establish successful infection. In contrast, boosting autophagy results in the clearance of intracellular bacteria and thereby inhibits infection. Small-molecule enhancers of autophagy are reported to control bacterial infections like *Mycobacterium* and *Salmonella* (Kim et al., 2012; Noad et al., 2017; Ammanathan et al., 2020). Recent studies have also focused on major autophagy players, such as mTOR, akt, and TFEB, a transcription factor controlling autophagy to reduce bacterial burden (Floto et al., 2007; Lin Y.-T et al., 2017). Hence, in this era of antimicrobial resistance, there is an urgent need to identify new antimicrobial compounds to overcome the problem of resistance mechanisms by targeting host factors like autophagy.

This study is the first evidence to show that capsaicin (Caps) induces autophagy for the intracellular killing of *S. flexneri*. Caps, a known anticancer herbal compound, is able to stimulate autophagy in intestinal cells and macrophages. Autophagy, in turn, inhibited the intracellular infiltration of *S. flexneri*. This is a novel approach in inhibiting *S. flexneri* infection. Previous reports indicate that Caps induces autophagy in tumor cells by overexpressing the autophagy proteins beclin1, Atg5, and LC3B (Jin et al., 2014). Consistent with these studies in cancer cells, we showed that Caps also augmented autophagy at a lower dose in intestinal epithelial cells at the protein level. This dose is nontoxic to cells, as reported earlier (Campbell-Valois et al., 2015). Further, we confirmed insignificant toxicity in HT29 cells and macrophages. Our findings showed for the first time that Caps treatment resulted in the overexpression of autophagy genes in intestinal cells. Unlike our knowledge of the overexpression of autophagy proteins by Caps, the transcriptional upregulation of autophagy by Caps has not been reported earlier. Furthermore, Caps induced autophagosome formation in intestinal and macrophage cells. Transcriptional activation of autophagy resulted in autophagosome formation.

In addition to autophagic stimulation, Caps attributed to the killing of *S. flexneri* in intestinal epithelial cells and macrophages. Along with bactericidal effects, our microscopic observations indicated autophagosome formation in Caps-treated cells. Electron and confocal microscopic studies revealed that bacteria are associated with autophagosomes in drug-treated cells. Consistently, we observed that Caps robustly increased LC3B puncta formation and targeted bacteria to autophagosome. In plasmid-cured avirulent strain of *S. flexneri* (lacking T3SS), Caps is unable to target bacteria, as most of the bacteria are outside the cells. Published reports have shown that the IcsB, VirA, and other proteins released by T3SS are required for the intracellular infiltration of *S. flexneri* (Ogawa et al., 2005; Vakifahmetoglu-Norberg et al., 2015). Hence, Caps targeted only intracellular bacteria. However, we checked whether Caps can directly kill the bacteria, but there is no direct effect of Caps on *S. flexneri*. Thus, it is confirmed that the anti-shigella effect of Caps is autophagy-dependent.

Previous reports of *S. flexneri* showed that Atg5, an essential autophagy protein, is needed for LC3 recruitment to *Shigella flexneri*. Here, we observed that the intracellular growth of *S. flexneri* remains unaffected due to the knockdown of Atg5 in intestinal cells. Caps treatment in Atg5 knockdown cells resulted in the loss of pathogen restriction, suggesting that autophagy plays an essential role in the inhibition of bacterial growth.

Having established that autophagy is the major player in Caps-mediated *S. flexneri* clearance, we explored the mechanistic details of autophagy induction by Caps. We observed that autophagic gene transcription is enhanced due to nuclear localization of TFEB (Sardiello, 2016). Drug treatment enhanced the nuclear translocation of TFEB in both infected and uninfected cells.

It has been reported that TFEB is a transcriptional regulator of autophagosomal biogenesis (Fan et al., 2018). It induces the transcription of several autophagosomal and lysosomal genes. Moreover, TFEB enhancers are currently reported for the development of therapeutics against intracellular pathogens (Ammanathan et al., 2020). Hence, it is a targeted transcription factor. Here, we observed for the first time that Caps induces TFEB binding to promoter elements to augment autophagic gene transcription.

Previous studies have shown that the depletion of TFEB induces the multiplication of pathogens by downregulating autophagy during infection (Gray et al., 2016). Similarly, in this study, TFEB knockdown in intestinal cells retained *S. flexneri* multiplication, and Caps treatment was unable to restrict bacterial growth, suggesting the importance of TFEB in controlling *S. flexneri* infection. In contrast, the overexpression of TFEB reduced bacterial growth. Therefore, TFEB is involved in Caps-mediated transcription of autophagy genes, resulting in the enhanced intracellular killing of *S. flexneri*. Moreover, capsaicin is known to selectively bind to the TRPV1 receptor and induce calcium influx (Liang et al., 2018; Allais et al., 2020). TRPV1 is expressed in intestinal cells and is known to induce autophagy (Chen et al., 2021; Wang et al., 2021). Hence, Caps-induced autophagy might be due to TRPV1 activation.

In the current study, we proved that the antimicrobial effect of Caps is mediated through autophagy. Moreover, we observed that Caps inhibited *S. flexneri* resistant strain growth at a higher dose. Thus, it is evident that probably Caps can address the problem of antimicrobial resistance in near future. Recent studies of *Salmonella* showed the effect of autophagy inducing compounds in mice (Ammanathan et al., 2020). Hence, we explored Caps’s effectivity in an *in vivo* mouse model. We observed that Caps increased the clearance of *S. flexneri* in intestinal tissues of infected mice. Here, we also showed that Caps treatment in *in vivo* mouse model induced autophagy. Thus, Caps is effective in preclinical models to boost autophagy and simultaneously decrease *S. flexneri* burden.

**FIGURE 8:**
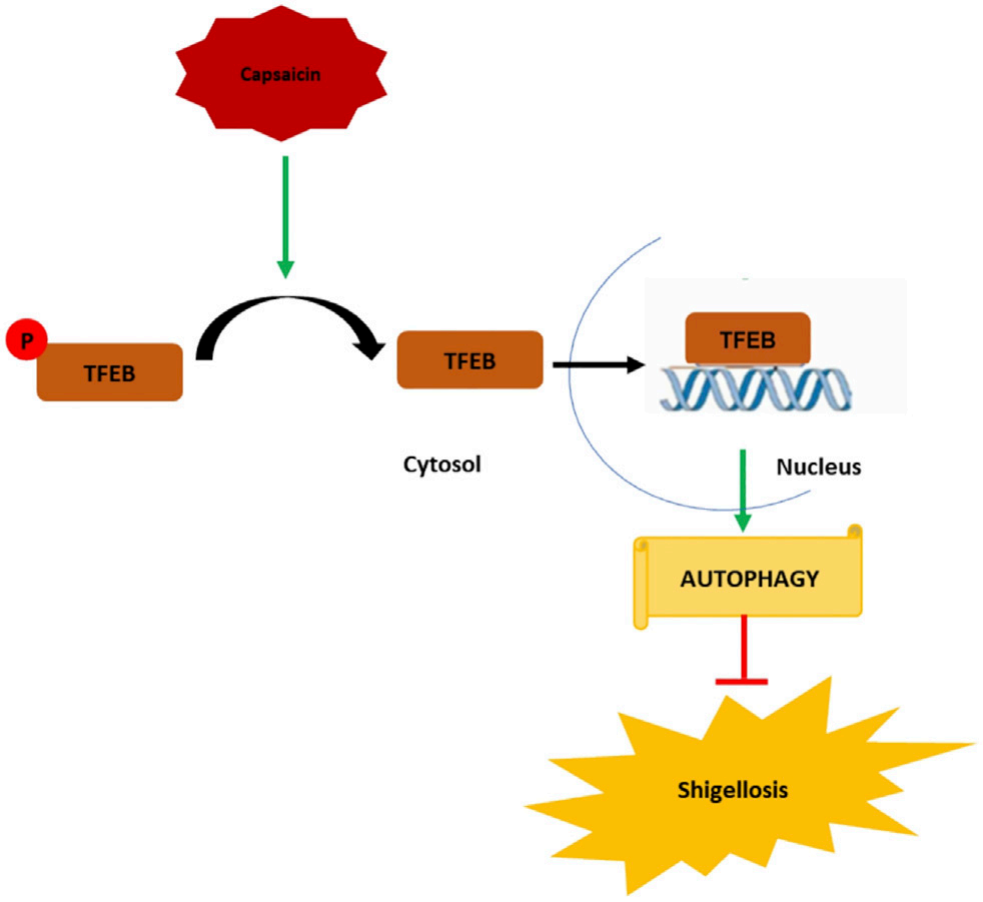
Graphical representation of the mechanism of action of capsaicin (Caps). Caps inhibits TFEB phosphorylation. Dephosphorylated TFEB moves from cytosol to nucleus and binds to the promoter site of autophagy genes resulting in the upregulation of autophagy-associated marker genes. Induction of autophagy inhibits shigellosis by inhibiting *S. flexneri* growth in both *in vitro* and *in vivo* models.

In conclusion, the emergence of antibiotic resistance requires a constant effort for the development of new therapies, which can boost host mechanisms to enhance bactericidal activity. Our data represent an important novel finding that drugs such as Caps manipulate host cellular defense mechanisms such as autophagy to achieve bactericidal effects against *S. flexneri*. The mechanistic details of Caps-activated autophagy uncovered a novel role of TFEB to be a potential target in treating *S. flexneri* infection. This is the first evidence of TFEB activation by Caps. Caps induced TFEB nuclear translocation by dephosphorylation of TFEB. TFEB in nucleus, in turn, binds to the promoter of autophagy genes to boost autophagy. Autophagy induction helps reduce *S. flexneri* growth and thereby inhibits shigellosis (Figure 8) Hence, a new alternative approach has been unraveled for the treatment of *S. flexneri* infection.

## 5 Conclusion

This study will open new avenues in treating shigellosis in the near future. More inducers of TFEB nuclear translocation, such as Caps, may be used to strengthen the host response against pathogens. However, Caps may not act as the only drug for the treatment of shigellosis, as most autophagy inducers are used in combination with other antibiotics for the treatment of bacterial pathogenesis. Host-directed therapy could be a new approach in adjunct to the use of antibiotics for infectious diseases. Moreover, Caps, as an autophagy inducer, might be effective against other intracellular pathogens.

## Data Availability

The original contributions presented in the study are included in the article/Supplementary Material; further inquiries can be directed to the corresponding author.
