# Supplementary material for: Capsaicin Inhibits Shigella flexneri Intracellular Growth by Inducing Autophagy
Source: Front Pharmacol. 2022 Jul 6;13:903438. doi: 10.3389/fphar.2022.903438 (PMC9298657; doi:10.3389/fphar.2022.903438)
Supplement: Supplementary file 1 [file DataSheet2.docx]

Supplementary Fig1


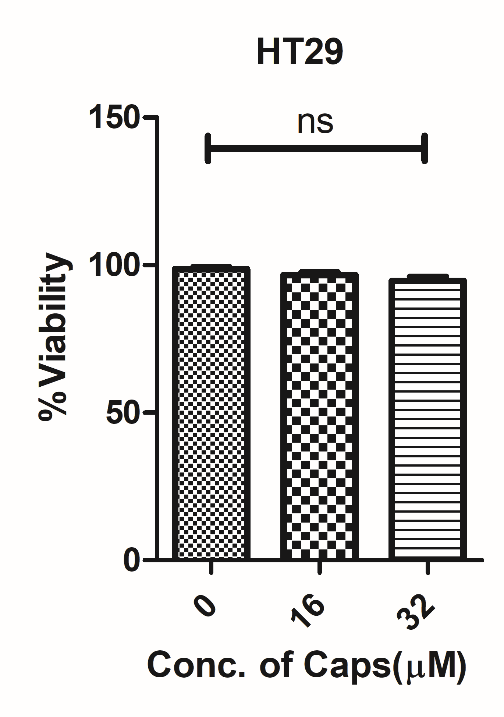

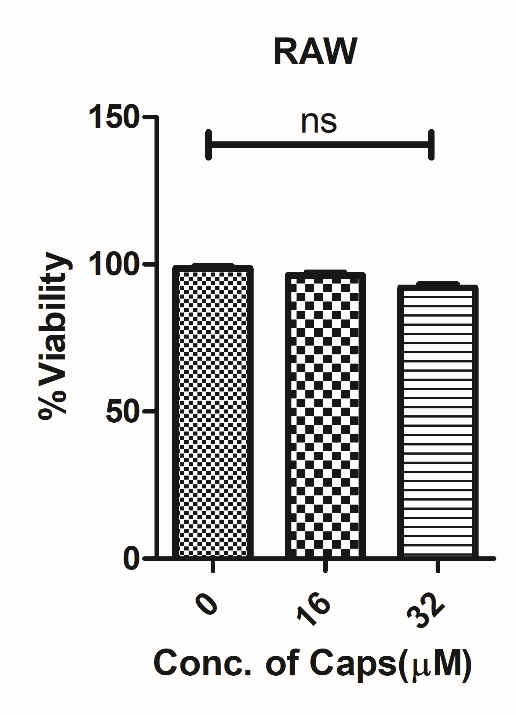


**FigS1:** HT29 and RAW 264.7 cells were treated with different concentration of Caps (16,32µM) or DMSO for 24 hrs. MTT assay was performed to measure % viability. One-way ANOVA was performed. Graphs were represented using GraphPad Prism 5 as mean±SEM (n=3);

Supplementary fig2


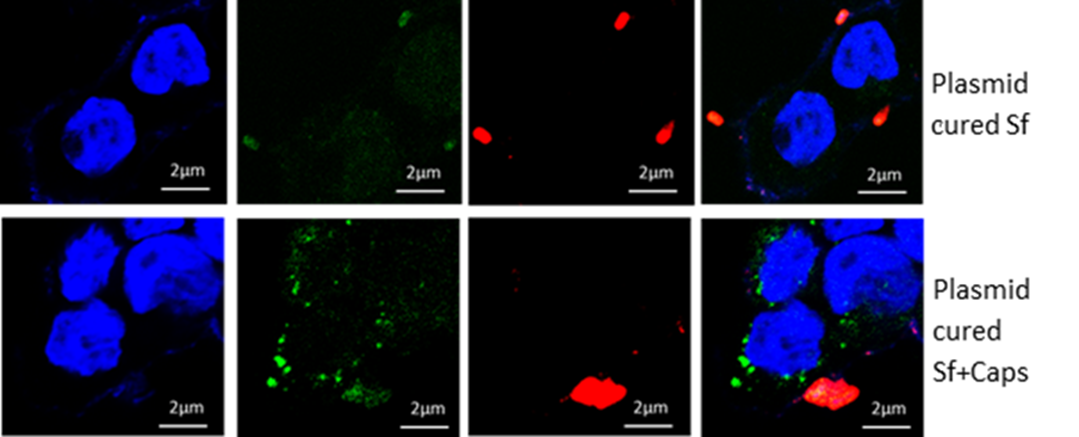


**FigS2:** Confocal microscopy of HT-29 cells infected with plasmid cured *S flexneri* (Red) and treated with Caps. LC3B (Green) puncta formation in GFP-LC3B overexpressed HT29 cells. Scale bar: 2μm.

Supplementary fig3


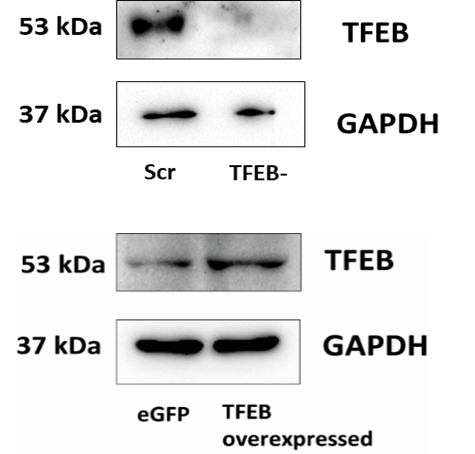


**FigS3**: Western blot showingTFEB expression in TFEB knockdown cells and scrambled siRNA cells. Western blot representing TFEB expression in eGFP-TFEB overexpressed cells. Gapdh was kept as control.

| **Name of Antibiotics** | **MIC (µg/ml)** | **MIC breakpoint of Resistance (CLSI guideline) µg/ml** |
| --- | --- | --- |
| Nalidixic Acid (NA) | 1250 | ≥8 |
| Ciprofloxacin (CIP) | 1250 | ≥1 |
| Norfloxacin (NOR) | 625 | ≥16 |
| Ofloxacin (OFX) | 2500 | ≥8 |
| Tetracyclin (TET) | 312 | ≥16 |
| Streptomycin (S) | 1250 | ≥32 |
| Chloramphenicol (C) | 1250 | ≥32 |
| Ampicillin (AM) | 312 | ≥32 |
| Erythromycin (E) | 1250 | ≥16 |
| Trimethoprim/ sulfamethoxazole (ST) | 1000 | ≥4 |
| Gentamicin (GEN) | 1 | ≥16 |

Supplementary Table1

**Supplementary Table 1:** *Shigella* *flexneri* (BCH12702) culture was treated with antibiotics (NA/CIP/NOR/OFX/TET/S/C/AM/E/ST) at different concentration in 96 well plate for 24hours and MIC was determined. Tabular representation shows the MIC (µg/ml) values and cut off for resistance according to CLSI guidelines.
