# Supplementary material for: Capsaicin Inhibits Shigella flexneri Intracellular Growth by Inducing Autophagy
Source: Front Pharmacol. 2022 Jul 6;13:903438. doi: 10.3389/fphar.2022.903438 (PMC9298657; doi:10.3389/fphar.2022.903438)
Supplement: Supplementary file 2 [file DataSheet1.docx]

Representative blots of Figure 2B


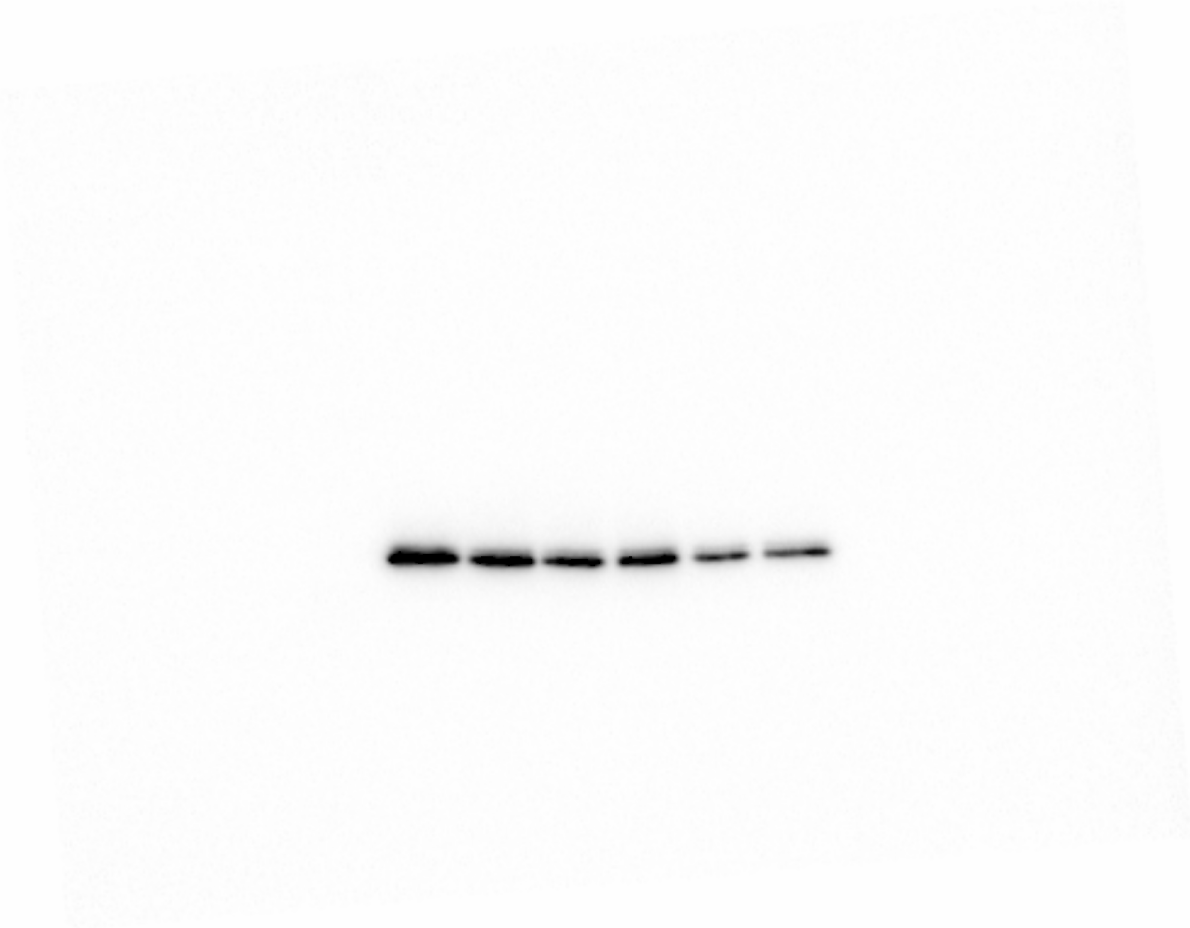


P62


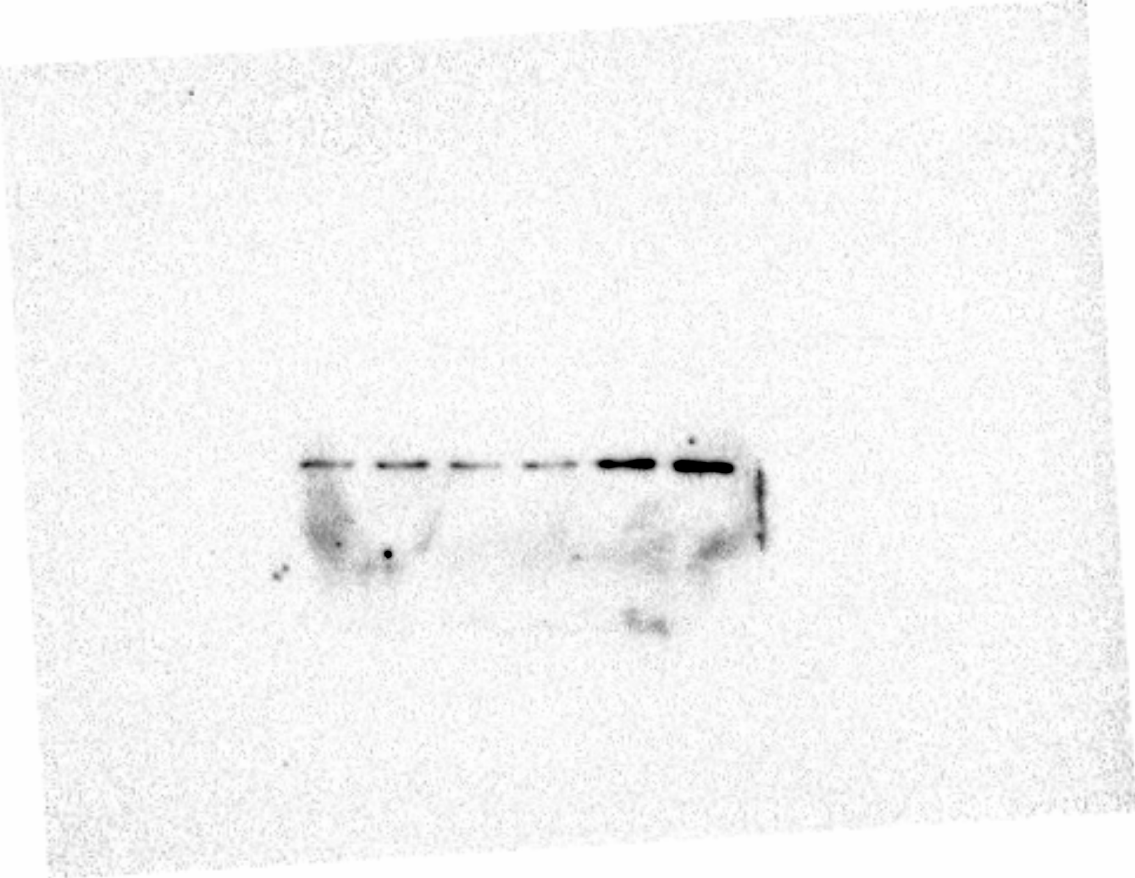


Atg5


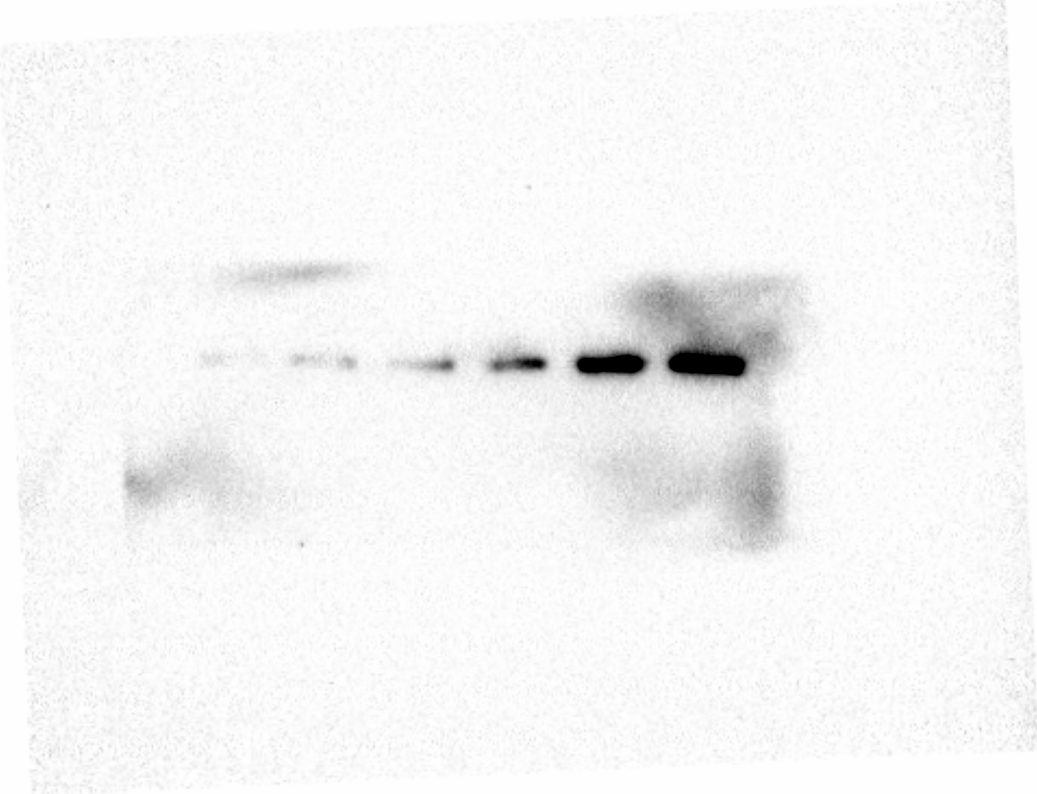


Beclin1


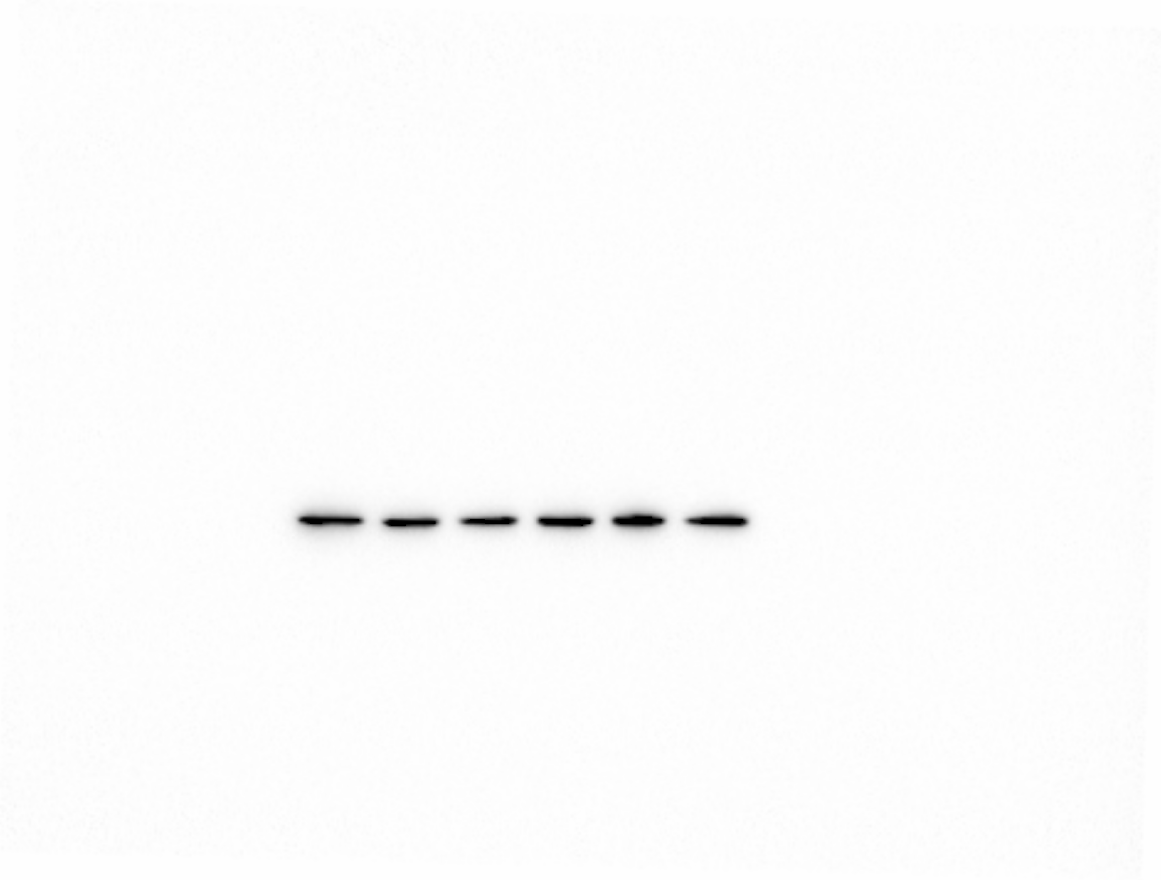


GAPDH

Representative blots of Figure 4D


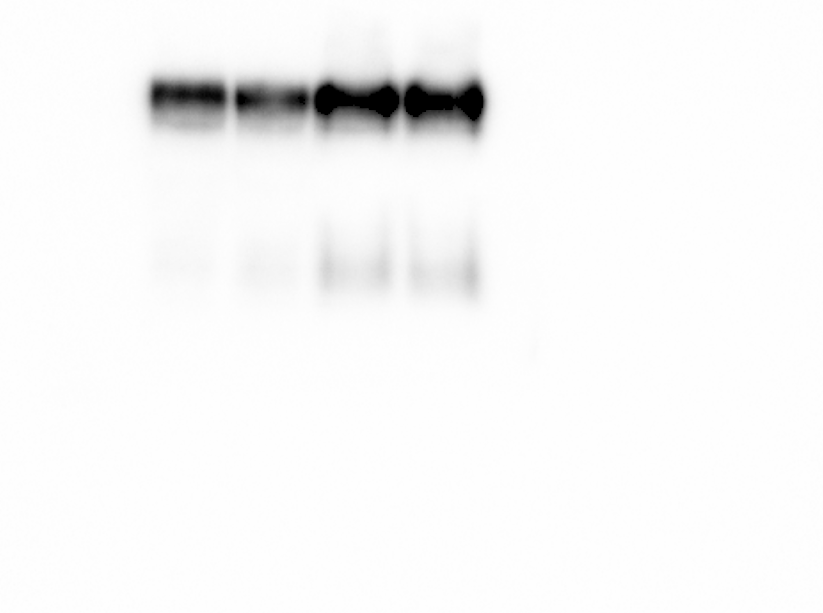


LAMP1


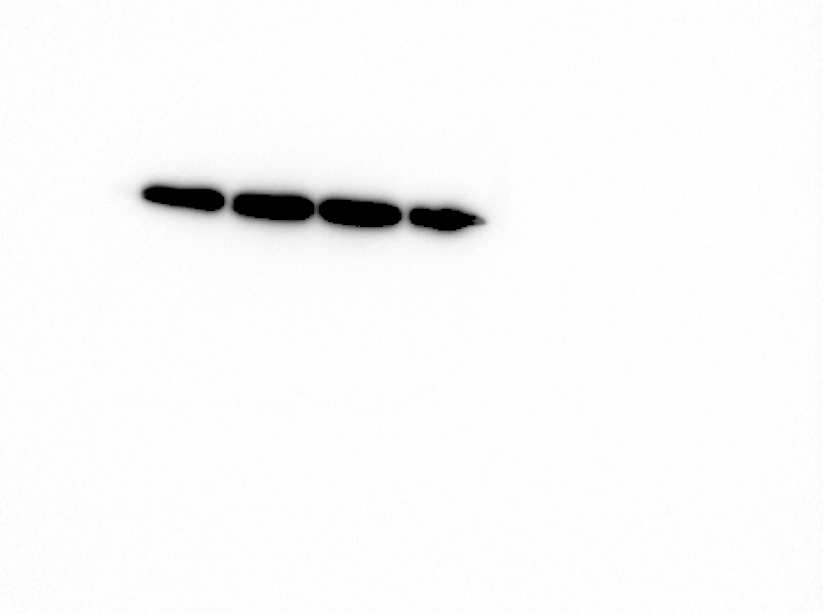


GAPDH


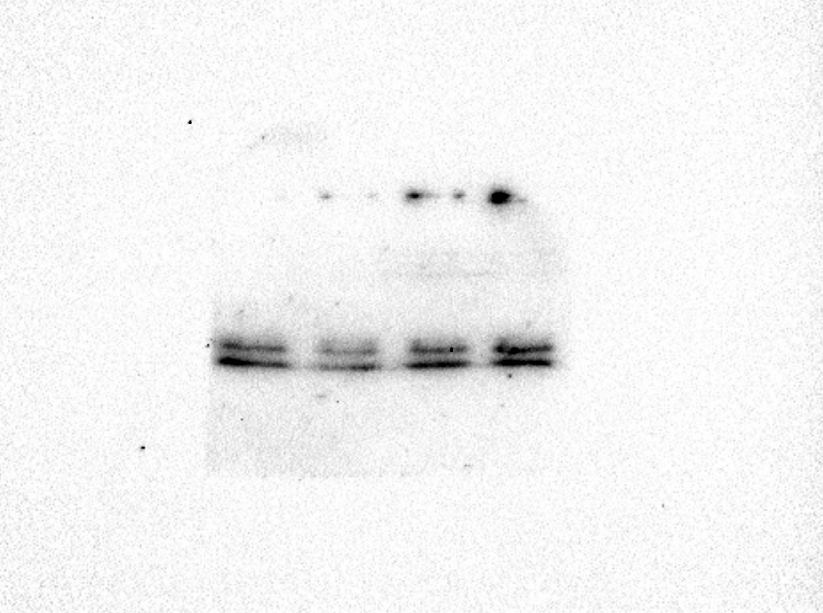


LC3B


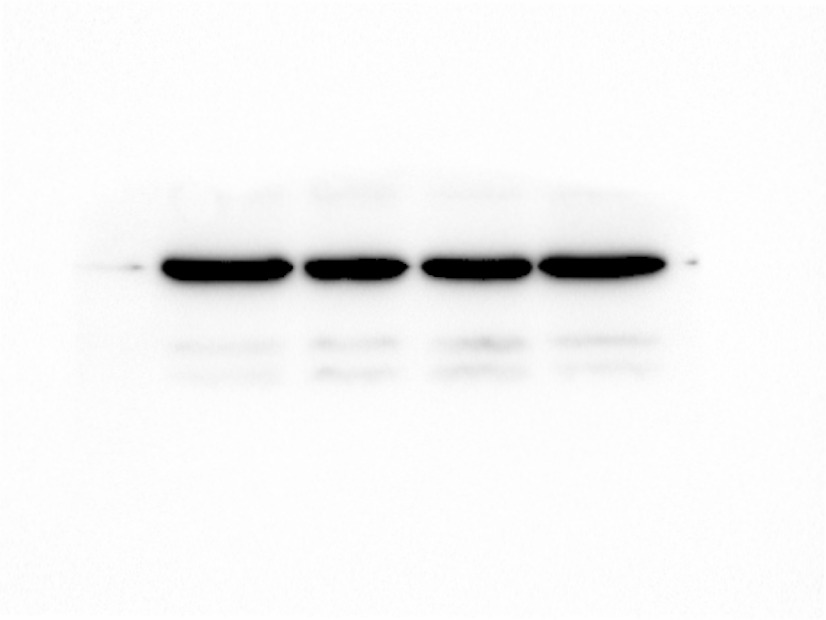


GAPDH

Representative blots of Figure 6D


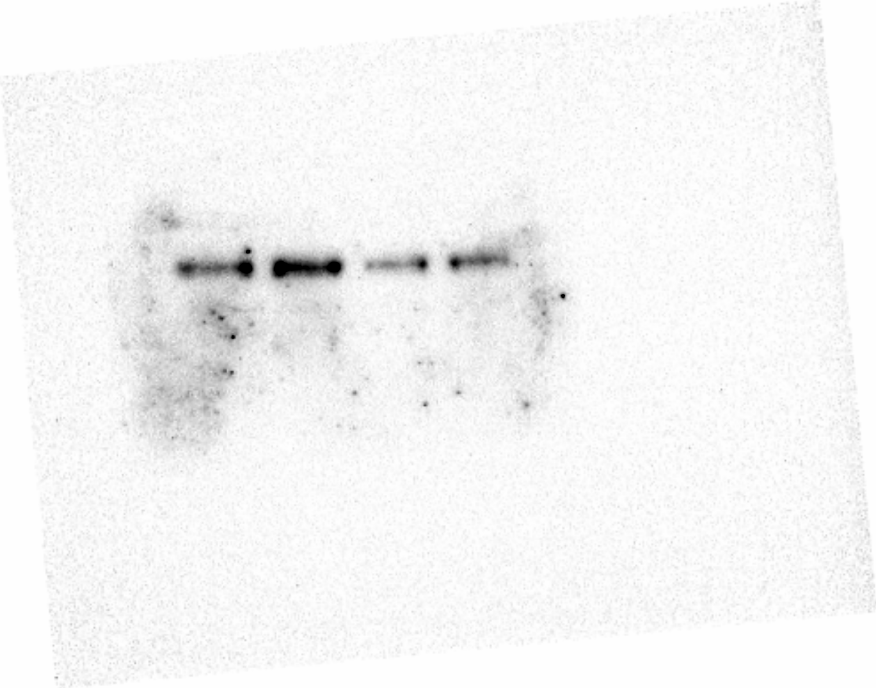


TFEB CE


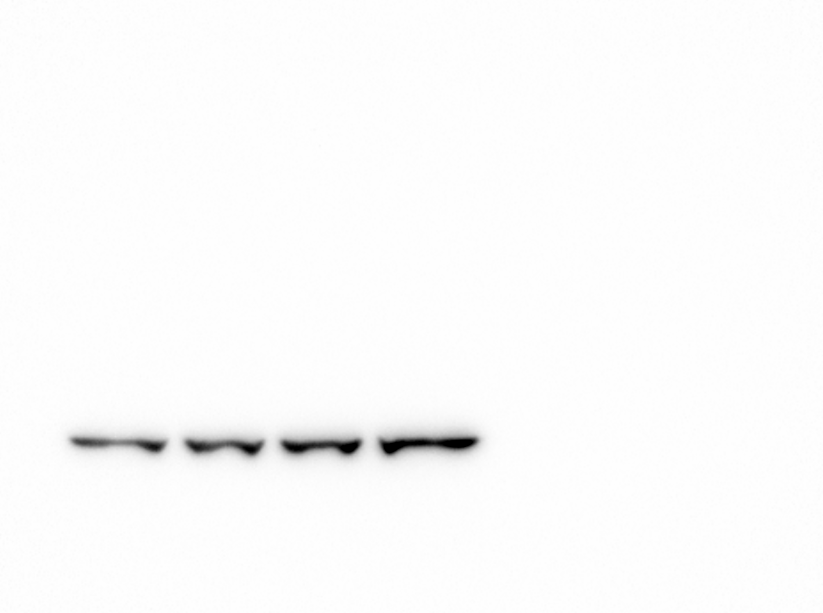


GAPDH


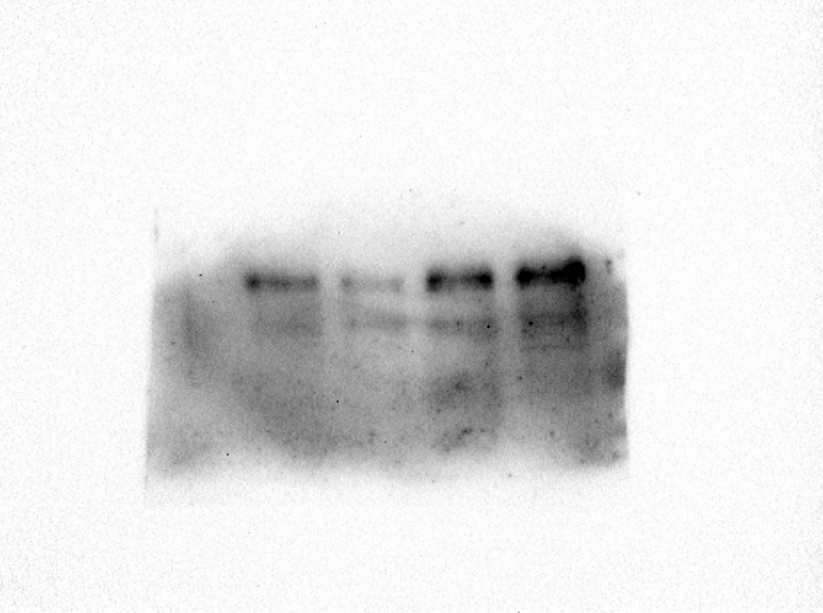

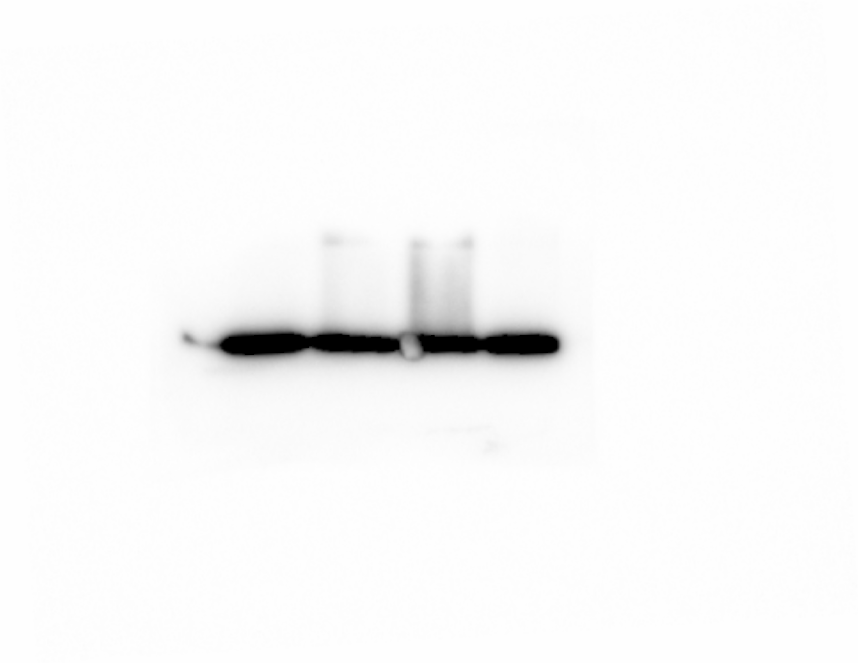


TFEB NE

Histone
